# Blueberry Bagasse-Enriched Whey Fermented Formulations: Effect of Incorporation Timing on Functional Properties and Neurobiological Evaluation in a Murine Model Using a Selected Formulation

**DOI:** 10.3390/nu18101558

**Published:** 2026-05-14

**Authors:** Tlalli Uribe-Velázquez, Alejandra Hurtado-Romero, Juliana Marisol Godínez-Rubí, Oscar Kurt Bitzer-Quintero, Javier Ramírez-Jirano, Félix Tadeo Ortiz-Sánchez, Jhonathan Cárdenas-Bedoya, Pablo Quintero-Gutiérrez, Iván Luzardo-Ocampo, Angélica Lizeth Sánchez-López, Luis Eduardo García-Amezquita, Danay Carrillo-Nieves, Tomás García-Cayuela

**Affiliations:** 1 Tecnologico de Monterrey, School of Engineering and Sciences, Ave. General Ramón Corona 2514, Zapopan 45138, Jal., Mexico; a01633168@tec.mx (T.U.-V.); ale.hr@tec.mx (A.H.-R.); a01644533@tec.mx (P.Q.-G.); ivanluzardo@tec.mx (I.L.-O.); alsl@tec.mx (A.L.S.-L.); danay.carrillo@tec.mx (D.C.-N.); 2 Laboratorio de Patología Diagnóstica e Inmunohistoquímica, Centro de Investigación y Diagnóstico de Patología, Departamento de Microbiología y Patología, Centro Universitario de Ciencias de la Salud, Universidad de Guadalajara, Guadalajara 44340, Jal., Mexico; juliana.godinez@academicos.udg.mx; 3 Instituto Mexicano del Seguro Social, Centro de Investigación Biomédica de Occidente, División de Neurociencias, Guadalajara 44340, Jal., Mexico; neuronim26@yahoo.com (O.K.B.-Q.); ramirez_jirano@hotmail.com (J.R.-J.); felixtadeo2010@gmail.com (F.T.O.-S.); jhonathan.cbedoya@academicos.udg.mx (J.C.-B.); 4 Departamento de Disciplinas Filósofico, Metodológicas e Instrumentales, Centro Universitario de Ciencias de la Salud, Universidad de Guadalajara, Guadalajara 44340, Jal., Mexico; 5 Tecnologico de Monterrey, Institute for Obesity Research, Ave. Eugenio Garza Sada 2501 Sur, Monterrey 64849, N.L., Mexico; 6 Tecnologico de Monterrey, School of Engineering and Sciences, Ave. Eugenio Garza Sada 2501 Sur, Monterrey 64849, N.L., Mexico; garcia-amezquita@tec.mx

**Keywords:** whey fermentation, blueberry bagasse, phenolic compounds, γ-aminobutyric acid, functional foods, probiotics, neurobiological effects

## Abstract

Background: Mental health disorders are a major global public health challenge. Psychobiotics have emerged as a promising strategy to modulate the microbiota–gut–brain axis. Whey and blueberry bagasse are agro-industrial by-products with potential for functional fermented matrices. Objective: To develop whey biotic blend (WBB) formulations enriched with blueberry bagasse and evaluate the impact of incorporation timing on functional properties, and to explore the neurobiological effects of a selected formulation. Methods: Three WBB formulations were prepared with *Lactiplantibacillus plantarum* 299v and PS128, differing in the timing of blueberry bagasse incorporation: Control, WBB-Before, and WBB-After. Physicochemical properties, microbial viability, composition, γ-aminobutyric acid (GABA), phenolic profile, and antioxidant and anti-inflammatory capacities were evaluated. Based on these results, WBB-After was selected as the sole formulation and advanced for testing in a lipopolysaccharide (LPS)-induced murine model. Results: All formulations supported probiotic growth (>8.6 log CFU/mL). Blueberry bagasse incorporation significantly influenced functional properties. WBB-After showed ~2.1-fold higher total phenolic content than WBB-Before and enrichment in anthocyanins and hydroxycinnamic acids, with higher antioxidant (≈24% by DPPH) and anti-inflammatory potential (≈19%), whereas WBB-Before exhibited ~20% higher GABA levels. In vivo, WBB-After showed improved recognition performance under baseline conditions (Control vs. WBB), although the overall group effect was marginal. No significant differences were observed in hippocampal cytokines or neuronal integrity markers under LPS-induced inflammation. Conclusions: The timing of blueberry bagasse incorporation shapes WBB functional properties. The selected formulation showed limited neurobiological effects under the evaluated in vivo conditions, highlighting the need for further studies.

## 1. Introduction

Mental health disorders represent a major global public health challenge, affecting nearly 1 in 7 people worldwide, with anxiety and depression being the most prevalent [1]. Despite the availability of psychotherapy and pharmacological interventions, access to treatment remains highly unequal, with a large proportion of the population lacking adequate care [2]. These limitations highlight the need for alternative and more accessible strategies to support mental health. In this context, growing evidence has highlighted the gut microbiota as a key modulator of brain function through the microbiota–gut–brain axis, a complex network linking the gastrointestinal tract and the central nervous system via neural, metabolic, endocrine, and immune pathways [3,4]. Dysbiosis has been associated with altered immune responses and increased inflammation, which have been linked to anxiety and depression [3,4]. Among emerging approaches targeting this axis, psychobiotics—defined as probiotic microorganisms that may confer mental health benefits—have gained increasing attention [5].

Fermented food matrices represent a promising and accessible vehicle for delivering psychobiotic microorganisms and their bioactive metabolites [6]. In particular, strains of *Lactiplantibacillus plantarum*, such as PS128 and 299v, have shown potential to modulate behavioral and neurochemical responses in both preclinical and clinical settings [7,8]. However, beyond strain selection, the role of formulation strategies in shaping the functional and neurobiological potential of fermented systems remains insufficiently explored.

Psychobiotics may influence the brain not only through the activity of live microorganisms but also through the production or modulation of neuroactive metabolites [6]. Among the key bioactive compounds produced by psychobiotics, γ-aminobutyric acid (GABA) has been associated with mental health-related processes. Within the central nervous system, GABA functions as the primary inhibitory neurotransmitter in charge of regulating processes such as anxiety, stress and sleep [9]. Certain gut bacteria, particularly lactic acid bacteria (LAB), can synthesize GABA as a defense against acidic stress [10], highlighting the relevance of fermented food systems as potential sources of this bioactive compound. However, beyond GABA, fermentation-derived substrates may contribute to the production of neuroactive compounds such as short-chain fatty acids, which have been linked to anti-inflammatory effects [3].

From a food systems perspective, whey represents a suitable and sustainable substrate for the development of fermented functional products. Whey is the primary by-product of the cheese industry and represents one of the largest waste streams generated during dairy processing [11]. Although whey contains approximately 90% water, it retains nearly 55% of the original nutritional components of milk, including lactose, proteins, lipids, and minerals, making it a valuable substrate for microbial fermentation processes [12]. Similarly, the growing demand for berries, driven by their association with a reduced risk of chronic diseases, has led to a parallel increase in berry processing and the generation of agro-industrial residues [13]. During juice or puree production, approximately 10–20% of the fruit mass is discarded as bagasse, composed mainly of skins, seeds, and residual pulp. Despite being considered a processing waste, berry bagasse retains significant concentrations of bioactive compounds, including phenolic acids, flavonoids, and anthocyanins, which are associated with antioxidant and antimicrobial activities [14]. Beyond their individual valorization, the interaction between agro-industrial by-products and fermentation processes is influenced by formulation strategies that can affect microbial metabolism, substrate availability, and phenolic compound transformation, thereby shaping the functional profile of the resulting fermented matrix [15,16,17]. In this context, the timing of incorporation of plant-derived substrates may play a key role, as compounds added before fermentation can undergo microbial transformation, whereas post-fermentation addition may favor their preservation. While previous studies have explored fermentation strategies to enhance the functional properties of blueberry and blueberry bagasse [18,19], the role of ingredient incorporation strategies during fermentation remains insufficiently explored, particularly in blueberry bagasse-based systems.

Many studies investigating psychobiotics have focused on either single probiotic strains or isolated bioactive compounds evaluated in simplified experimental systems [7,8,20]. However, the potential synergistic effects of integrating fermented dairy substrates, specific psychobiotic candidate strains, and polyphenol-rich agro-industrial by-products remain poorly understood. In particular, limited attention has been given to how these formulation strategies during fermentation influence the resulting bioactive profile and its functional properties. Furthermore, while numerous studies report in vitro bioactivities, there is still a lack of integrative approaches that combine detailed compositional characterization with in vivo evaluation of neurobiological outcomes. This gap highlights the need for studies that bridge the relationship between fermentation-derived bioactive compounds and their potential physiological effects in complex biological systems.

We hypothesize that the timing of blueberry bagasse incorporation during fermentation modulates the generation of bioactive compounds and the functional properties of the formulations, with potential implications for neurobiological outcomes.

To address this, the aims of this study were (i) to develop and characterize whey-based fermented formulations enriched with blueberry bagasse and to evaluate how incorporation timing influences their functional properties, and (ii) to perform an exploratory in vivo evaluation of the selected formulation. Two formulations were produced depending on the stage of bagasse incorporation (before or after fermentation) and were characterized in terms of physicochemical properties, nutritional composition, GABA production, phenolic profile, and in vitro antioxidant and anti-inflammatory activities. Based on these results, a selected formulation was further evaluated in a murine model to assess behavioral responses, neuronal integrity, and inflammatory cytokine levels.

## 2. Materials and Methods

### 2.1. Preparation of Whey and Blueberry Bagasse Ingredients

Whey was prepared under controlled laboratory conditions using commercial whole milk (Grupo Lala, Gómez Palacio, Mexico) to ensure process standardization. The milk was poured into a stainless-steel container and heated to 35–38 °C. Then, 0.15 mL of rennet (Cuamex, Ciudad de México, Mexico) per liter of milk and 15 mg of calcium chloride per liter were added. The mixture was incubated at 32 °C for 45 min, after which the curd was cut, and the whey was separated by filtration. The whey was subsequently pasteurized at 72 °C for 15 s. Following pasteurization, samples were cooled, stored at 4 °C under aseptic conditions, and used within 24 h. These conditions ensured microbiological stability.

The blueberries (*Vaccinium corymbosum*, ‘Biloxi’ variety) used were obtained from a local grocery store in Guadalajara, Mexico, and stored at −20 °C until use. Blueberry bagasse was obtained as described previously [14]. Briefly, thawed blueberries were blended and centrifuged to separate the juice from the solid fraction (bagasse). The resulting bagasse was lyophilized, ground, sieved (105 μm), and stored at −40 °C until further use.

### 2.2. Preparation of Whey Biotic Blend (WBB) Formulations

The whey biotic blend (WBB) formulations were prepared using pasteurized whey obtained as described in Section 2.1. Fermentation was carried out using two commercial probiotic strains, *L. plantarum* 299v and *L. plantarum* PS128, which were previously maintained as cryopreserved stocks at −80 °C. Prior to fermentation, each strain was reactivated in the de Man, Rogosa, and Sharpe (MRS) broth and incubated at 37 °C for 24 h. Cultures were then harvested by centrifugation (3000× *g*, 10 min, 4 °C), washed twice, and resuspended in sterile saline solution (0.85%, *w*/*v*). Under these conditions, cultures typically reached viable counts of 8.7–8.9 log colony-forming units per mL (CFU/mL).

Pasteurized whey was supplemented with sodium glutamate (5 mM) as a precursor for GABA synthesis and inoculated with both strains at 2% (*v*/*v*) each. Fermentation was carried out at 37 °C for 48 h.

Three WBB formulations were obtained depending on the stage at which blueberry bagasse was incorporated, to evaluate the effect of incorporation timing (before fermentation to allow microbial transformation vs. after fermentation to favor compound preservation). The control formulation consisted of fermented whey without blueberry bagasse (WBB-Control). In the co-fermented formulation (WBB-Before), freeze-dried blueberry bagasse was added to the whey prior to fermentation at a final concentration of 2.5% (*w*/*v*), and the mixture was vortex-mixed to ensure proper dispersion and fermented under the same conditions described above. In the third formulation (WBB-After), whey was first fermented under the previously described conditions and subsequently supplemented with freeze-dried blueberry bagasse at a final concentration of 2.5% (*w*/*v*), followed by vortex mixing. All formulations were vortex-mixed prior to analysis to ensure consistent sample handling across treatments.

Microbial counts in the fermented samples were performed by serial decimal dilutions in sterile saline solution, followed by spread plating on MRS agar. Plates were incubated at 37 °C for 48 h, and results were expressed as log CFU/mL. Initial counts after inoculation ranged from 6.6 to 6.9 log CFU/mL across all formulations.

### 2.3. Physicochemical Characterization

The pH of the samples was measured using a pH meter (LAQUAact-PH110-K, HORIBA, Kyoto, Japan). Titratable acidity, expressed as g of lactic acid/100 g of fresh weight, was determined by titration with 1 mol/L NaOH until reaching pH 8.3, following the Mexican Official Standard NMX-155-SCFI-2012 [21]. Color parameters were measured using a spectrophotometer (Minolta Co., Kyoto, Japan), according to the Commission Internationale de l’Èclairage (CIE) color system, recording L*, a*, and b* values corresponding to lightness, red–to-green, and yellow–to-blue coordinates, respectively.

### 2.4. Proximate Composition

Proximate composition was determined according to standardized methods based on Mexican official standards (NOM/NMX) and AOAC procedures, and results were expressed on a wet basis. Moisture content was determined by drying samples to constant weight (NOM-116-SSA1-1994) [22]. Ash content was measured by incineration at 550 °C until constant weight (NMX-F-607-NORMEX-2020) [23]. Total lipids were determined by ether extraction (NOM-086-SSA1-1994) [24], while crude protein was measured using the Kjeldahl method (NMX-F-608-NORMEX-2011) [25]. Total carbohydrates were calculated by difference as 100 minus the sum of moisture, ash, protein, and lipids. Additionally, dietary fiber content (including soluble and insoluble fractions) was determined using the AOAC method 2011.25, with minor modifications as previously described [26].

### 2.5. Phenolic Compounds and Functional Properties

#### 2.5.1. Preparation of Extracts for Phenolic and Functional Analyses

Samples were freeze-dried, and aqueous methanolic extracts (50%, *v*/*v*) were prepared using 1 g of freeze-dried powder as previously described [27].

#### 2.5.2. Total and Individual Phenolic Compounds

Total phenolic content (TPC) was determined using the Folin–Ciocalteu assay as previously described [28]. Absorbance was measured at 765 nm using a microplate reader (Varioskan Lux, Thermo Fisher Scientific, Waltham, MA, USA), and results were expressed as mg of gallic acid equivalents (GAE) per g of dry weight (d.w.).

Individual phenolic compounds were analyzed using an ultra-high-performance liquid chromatography (UHPLC) system using an Acquity Arc system equipped with a photodiode array detector (Waters, Milford, MA, USA) and a reverse-phase C18 column (4.6 mm × 150 mm, 2.7 µm; Cortecs, Waters) maintained at 40 °C. Chromatographic separation was performed as previously described [18]. The mobile phase consisted of 1% (*v*/*v*) formic acid in water (solvent A) and 1% (*v*/*v*) formic acid in methanol (solvent B). Elution was carried out at a flow rate of 0.6 mL/min with an injection volume of 7 µL using a gradient program (B, %): 13.5% (0–8 min), 18% (8–12 min), 30% (12–20 min), 35% (20–23 min), 65% (23–30 min), and 22.5% (30–33 min), followed by re-equilibration to initial conditions. Detection was performed at 280, 360, and 520 nm to monitor different classes of phenolic compounds. Compounds were identified by comparing retention times and UV–Vis spectra with those of authentic standards and literature data, and quantified using external calibration curves. Results were expressed as µg/g d.w.

#### 2.5.3. Antioxidant and Anti-Inflammatory Activities

Antioxidant activity was evaluated using the 2,2-diphenyl-1-picrylhydrazyl (DPPH) and 2,2′-azinobis(3-ethylbenzothiazoline-6-sulfonic acid) (ABTS) radical scavenging assays as previously described [29]. Absorbance was measured at 517 nm (DPPH) and 734 nm (ABTS) using a microplate reader, and results were expressed as mg of Trolox equivalents (TE) per g of d.w.

Nitric oxide radical scavenging activity (NO-RSA) was determined using the Griess–Ilosvory reaction as previously described [30], with minor modifications. Briefly, samples were incubated with sodium nitroprusside to generate nitric oxide, followed by reaction with Griess reagents. The total reaction volume was adjusted to 2 mL, and the sample volume was 250 µL. Absorbance was measured at 540 nm using a microplate reader, and results were expressed as mg TE per g of d.w.

#### 2.5.4. GABA Quantification

GABA was quantified using a GABase enzymatic assay (Sigma-Aldrich, St. Louis, MO, USA) as previously described [31]. Briefly, fresh samples were centrifuged and filtered (0.45 µm), and 10 µL of the clarified supernatant was mixed with reaction buffer in a 96-well microplate. The reaction was incubated at 30 °C, and NADPH formation was measured at 340 nm using a microplate reader. GABA concentrations (mM) were calculated using an external calibration curve.

### 2.6. Animals and Experimental Design

Male and female Sprague Dawley rats (60 days old) were obtained from the Centro de Investigación Biomédica de Occidente (CIBO; Guadalajara, Mexico). Animals were housed in polycarbonate cages (6 animals per cage) under controlled environmental conditions (24 ± 1 °C, 50 ± 5% humidity) with a 12 h light–dark cycle. Standard laboratory chow and water were provided ad libitum throughout the experimental period. Animals were randomly assigned to four experimental groups (*n* = 20 per group): (1) control group receiving water, (2) WBB group receiving the WBB-After formulation, (3) LPS group receiving water followed by lipopolysaccharide (LPS; 15 mg/kg), a dose consistent with endotoxic shock models (LD75) to induce acute systemic inflammation with neurobiological effects [32], and (4) WBB + LPS group receiving the WBB-After formulation followed by LPS administration.

All treatments were administered daily by oral gavage (1 mL per animal) for 21 consecutive days. The in vivo phase was designed as an exploratory evaluation of the selected formulation following in vitro characterization. Accordingly, the administered WBB-After dose corresponded to the composition of the formulation as obtained after fermentation and supplementation, providing approximately 4.6 × 10^8^ CFU of probiotics, 201 µg of GABA, and 25 mg of blueberry bagasse per animal per day.

LPS was administered on day 20 to induce an acute neuroinflammatory challenge, allowing the evaluation of the potential preventive effects of dietary intervention. Animals were further subdivided according to the experimental endpoint (behavioral analysis, histopathological studies or molecular analyses), maintaining sex-balanced subgroups.

All animals were clinically healthy at the start of the study and had not been used in previous experimental procedures. Animals were monitored throughout the experimental period, particularly following LPS administration. Humane endpoints were predefined, and criteria for early euthanasia included signs of severe distress or compromised condition. No animals met these criteria, and no unexpected adverse events were observed during the study.

### 2.7. Behavioral Assessment

Cognitive performance was evaluated using the novel object recognition (NOR) test as previously described [33]. Behavioral testing was conducted at the end of the treatment period in an open-field arena (50 cm × 50 cm × 50 cm) with black acrylic walls under dim lighting conditions. The NOR protocol consisted of three phases. During habituation, each rat was allowed to explore the empty arena for 10 min. Twenty-four hours later, the familiarization phase was performed by allowing animals to explore two identical objects for 5 min. After a 1 min interval, the test phase was conducted by replacing one of the familiar objects with a novel object and allowing exploration for 5 min. Exploration was defined as direct sniffing or close interaction with the objects. Recognition memory was assessed using the discrimination index, defined as the proportion of time spent exploring the novel object relative to total object exploration. To minimize bias, objects were cleaned with 70% ethanol, and bedding was rearranged between trials.

### 2.8. Tissue Collection and Processing

To evaluate neuronal death, six animals from each experimental group were deeply anesthetized with a combination of xylazine (5 mg/kg body weight) and ketamine (50 mg/kg body weight) and transcardially perfused. Perfusion was initiated with phosphate-buffered saline (PBS; 0.1 M, pH 7.4) supplemented with sodium heparin (1000 IU/L) as an anticoagulant and procaine hydrochloride (1 g/L) as a vasodilator, followed immediately by perfusion with 200 mL of 4% paraformaldehyde prepared in PBS. After perfusion, a craniotomy was performed, and the brains were carefully removed and post-fixed in 4% paraformaldehyde in PBS.

### 2.9. Histological and Immunohistochemical Analyses

#### 2.9.1. Hematoxylin and Eosin Staining

Hematoxylin and eosin (H&E) staining was performed on formalin-fixed, paraffin-embedded (FFPE) tissue sections using standard histological procedures. Briefly, coronal brain sections containing the hippocampal CA1 region were cut at a thickness of 5 µm, mounted on glass slides, and dried prior to staining. Sections were deparaffinized in xylene and rehydrated through a graded ethanol series to distilled water. Slides were then stained with hematoxylin and eosin to visualize cellular morphology, followed by dehydration through ascending ethanol concentrations, clearing in xylene, and coverslipping with mounting medium. Stained slides were allowed to dry prior to digital scanning for quantitative histological analysis.

#### 2.9.2. Immunohistochemistry

Immunohistochemistry (IHC) was performed on FFPE tissue sections following deparaffinization and rehydration as described above, using the ImmPRESS^®^ Excel Amplified Polymer Kit Peroxidase (Anti-Rabbit IgG, Vector Laboratories, Newark, CA, USA), according to the manufacturer’s instructions with minor modifications. Antigen retrieval was carried out using heat-induced epitope retrieval (HIER) in Tris-EDTA buffer (pH 9.0). Sections were incubated with the primary antibody (rabbit anti-NeuN, Abcam, Cambridge, UK; cat. ab177487; dilution 1:100) for 2 h at room temperature. Slides were then processed following the kit protocol, counterstained with hematoxylin for 10 min, dehydrated, cleared in xylene, and mounted using Entellan Neu mounting medium (Merck KGaA, Darmstadt, Germany; cat. 1.00869). Appropriate positive and negative controls were included, and negative controls were prepared by omitting the primary antibody. NeunN-positive cells were quantified in the hippocampal CA1 region.

#### 2.9.3. Detection of Apoptosis by TUNEL Assay

Terminal deoxynucleotidyl transferase dUTP nick end labeling (TUNEL) staining was performed on FFPE tissue sections using the Click-iT™ Plus TUNEL Assay (Thermo Fisher Scientific, Waltham, MA, USA; cat. C10617/C10618/C10619) according to the manufacturer’s protocol. Briefly, tissue sections were deparaffinized in xylene and rehydrated through a graded ethanol series, followed by permeabilization with proteinase K. Sections were then incubated with the terminal deoxynucleotidyl transferase (TdT) reaction mixture to incorporate EdUTP into DNA strand breaks. After washing, slides were incubated with the Click-iT™ Plus TUNEL reaction cocktail containing Alexa Fluor™ 594 picolyl azide dye for fluorescent detection of fragmented DNA via copper-catalyzed click chemistry. Subsequently, sections were immunostained with a primary anti-NeuN antibody (EPR12763) to identify neuronal cells, followed by incubation with a goat anti-rabbit IgG (H+L) cross-adsorbed secondary antibody conjugated to Alexa Fluor™ 488 (green). Nuclei were counterstained with 4′,6-diamidino-2-phenylindole (DAPI), and samples were imaged under a fluorescence microscope. Positive and negative controls were included according to the manufacturer’s recommendations. TUNEL-positive cells and their co-localization with NeuN were quantified in the hippocampal CA1 region.

#### 2.9.4. Image Acquisition and Quantitative Analysis

Histological and immunohistochemical slides were digitized using an Aperio LV1 IVD whole-slide scanner (Leica Biosystems, Wetzlar, Germany). The resulting images were analyzed using QuPath software (version 0.4.3), an open-source platform for digital pathology and whole-slide image analysis [34]. Regions of interest (ROIs) corresponding to the hippocampal CA1 region were manually delineated based on anatomical landmarks defined in the Paxinos and Watson rat brain atlas [35] and supported by an interactive digital atlas [36]. Analyzed sections spanned approximately from −2.2 to −5.3 mm posterior to bregma. Whenever possible, H&E, immunohistochemical, and TUNEL analyses were performed on adjacent or closely matched sections to ensure evaluation of comparable anatomical regions. Automated cell detection and classification tools in QuPath were used to identify and quantify positively stained cells within the defined ROIs. Detection thresholds were established based on signal intensity above background levels. Cell density was calculated as the number of positively stained cells per square millimeter (cells/mm^2^) within the defined ROIs. For immunofluorescence analyses, images were acquired using a Carl Zeiss Axio Imager A2 fluorescence microscope (Zeiss, Oberkochen, Germany) and processed using Zeiss ZEN software (version 3.8). Fluorescence images were subsequently analyzed using QuPath for quantitative assessment.

### 2.10. Cytokine Quantification

Cytokine levels, including interleukin-1β (IL-1β), tumor necrosis factor-α (TNF-α), interleukin-6 (IL-6), and interleukin-10 (IL-10), in hippocampal tissue were determined using a MILLIPLEX MAP Rat Cytokine/Chemokine Magnetic Bead Panel-Immunology Multiplex Assay (Millipore, Merck KGaA, Darmstadt, Germany; cat. RECYTMAG-65K) according to the manufacturer’s instructions.

Hippocampi were collected immediately after decapitation, homogenized in ice-cold PBS containing a protease inhibitor cocktail (Calbiochem-Novabiochem, Merck Millipore, San Diego, CA, USA), and centrifuged to obtain clarified supernatants. Protein concentration was determined using a NanoDrop 2000 spectrophotometer (Thermo Fisher Scientific, Waltham, MA, USA) at 280 nm, and samples were adjusted to 4 mg/mL by dilution with PBS. Cytokine quantification was performed following the standard multiplex immunoassay workflow, including incubation with antibody-coated magnetic beads, detection antibody labeling, and signal development with streptavidin–phycoerythrin. Samples were analyzed using a Luminex^®^ xMAP^®^ system (Luminex Corporation, Austin, TX, USA), and cytokine concentrations were calculated from median fluorescence intensity using a five-parameter logistic standard curve. Cytokine levels were expressed as pg/mg protein.

### 2.11. Statistical Analysis

All experiments related to formulation development and in vitro characterization were conducted using three independent biological replicates, with each analysis performed at least in triplicate. For in vivo experiments, the individual animal was considered the experimental unit. Behavioral assessments were performed independently for each animal. For histological analyses, quantitative measurements were obtained from ROIs within the hippocampal CA1 region in representative sections from each animal. Cytokine quantification was performed using hippocampal samples obtained from each individual animal.

Normality and homogeneity of variances were assessed prior to analysis, using Shapiro–Wilk’s test and Bartlett/Levene test for homoscedasticity. Differences among experimental groups were evaluated using one-way analysis of variance (ANOVA) followed by Tukey’s multiple comparison post hoc test when data met normality and homogeneity of variance assumptions. When heterogeneity of variances was detected, Welch’s ANOVA followed by Dunnett’s post hoc test (for comparisons against the control group) was applied. For data not meeting parametric assumptions, a Kruskal–Wallis test followed by Dunn’s multiple comparison test was used. For comparisons involving two groups, Student’s *t*-test for independent samples was used. Statistical significance was set at *p* < 0.05. Statistical analyses were carried out using OriginPro 2026 (OriginLab Corporation, Northampton, MA, USA). Data visualization was performed using Python 3.12.3 (NumPy 1.26.4, Pandas 2.1.4, and Matplotlib 3.6.3 libraries).

## 3. Results

### 3.1. Microbial Viability, Physicochemical Properties, and Proximate Composition of WBB Formulations

Microbial viability, physicochemical properties, and proximate composition of the formulations are summarized in Table 1. Microbial viability after fermentation did not differ significantly among formulations (8.7–9.0 log CFU/mL), representing an increase from the initial inoculum levels in whey.

The pH decreased significantly after fermentation, with all samples reaching values below 4. Among formulations, WBB-Before and WBB-After exhibited lower pH values than the control. Titratable acidity followed an inverse trend, with higher values in WBB-Before and lower values in WBB-After. Soluble solids increased with the incorporation of blueberry bagasse, reaching the highest values in WBB-After. Color parameters differed among formulations, with WBB-Control showing higher lightness, whereas bagasse-containing samples were darker and shifted toward red tones. Differences in b* values indicated a transition from positive (yellow) in the control to lower or negative values in enriched formulations.

Proximate composition reflected the liquid nature of the samples, with moisture contents above 90% in all cases. Ash content was higher in WBB-Control and decreased in bagasse-enriched samples. Protein levels were slightly higher in WBB-Before and WBB-After, while fat and total carbohydrate contents showed only minor variations. As expected, dietary fiber was only detected in WBB-Before and WBB-After (~1.2%), predominantly as insoluble fiber, reflecting the incorporation of blueberry bagasse.

### 3.2. Phenolic Composition of WBB

The phenolic composition of WBB formulations was primarily driven by the incorporation of blueberry bagasse; therefore, phenolic characterization focused on WBB-Before and WBB-After samples. TPC differed significantly between formulations, with WBB-After showing 2.1-fold higher values than WBB-Before, indicating that the timing of bagasse incorporation markedly influenced the overall phenolic content (Table 2).

A total of 29 phenolic compounds were identified by UHPLC (Table S1 and Figures S1 and S2), including hydroxybenzoic acids, hydroxycinnamic acids, flavan-3-ols, flavonols, and anthocyanins. For quantification purposes, dimeric and oligomeric proanthocyanidins were grouped and reported as a single class, resulting in 25 quantified entries (Table 2). No phenolic compounds were detected in the control formulation, confirming that the phenolic profile of WBB samples was exclusively associated with the incorporation of blueberry bagasse.

The stage at which bagasse was incorporated had a marked effect on phenolic composition. WBB-Before exhibited a profile enriched in flavan-3-ols and flavonols, whereas WBB-After showed a clear shift toward anthocyanins and hydroxycinnamic acids. In WBB-Before, grouped proanthocyanidins were predominant, with higher levels than in WBB-After (≈344 vs. ≈29 µg/g d.w.). Flavonols also contributed substantially, with quercetin derivatives and myricetin glycoside representing major components. In contrast, WBB-After was characterized by a pronounced enrichment in anthocyanins, with compounds such as peonidin-3-glucoside and malvidin-3-glucoside showing strong increases (up to ≈491 and ≈294 µg/g d.w., respectively), along with a notable rise in chlorogenic acid (≈428 vs. 35 µg/g d.w.). Differences were also observed within hydroxybenzoic acids, with gallic acid detected only in WBB-Before, while syringic acid was significantly higher in WBB-After. Overall, these differences in phenolic composition may contribute to variations in the functional properties of the formulations.

### 3.3. Functional Properties of WBB

The incorporation of blueberry bagasse markedly enhanced the functional properties of WBB formulations (Table 3). As expected, the control showed low antioxidant capacity, whereas both WBB-Before and WBB-After exhibited substantially higher values. ABTS levels were similar between WBB-Before and WBB-After, while DPPH showed a clearer differentiation, with WBB-After presenting the highest activity (≈3.8 mg TE/g d.w.).

The anti-inflammatory potential, assessed by NO-RSA, was not detected in the control formulation and increased markedly with bagasse incorporation, with WBB-After showing the highest activity (≈17 mg TE/g d.w.), approximately 20% higher than WBB-Before.

GABA concentration also differed significantly among formulations, with WBB-Before showing slightly higher levels (≈2.35 mM) than WBB-After (≈1.95 mM), and both markedly higher than the control (≈0.4 mM).

Overall, both formulations exhibited relevant functional attributes. However, WBB-After showed higher phenolic content and enhanced antioxidant and anti-inflammatory capacities while maintaining substantial GABA levels, and was therefore selected for in vivo evaluation as a multi-component functional matrix.

### 3.4. Behavioral Assessment (Novel Object Recognition Test)

**Figure 1:**
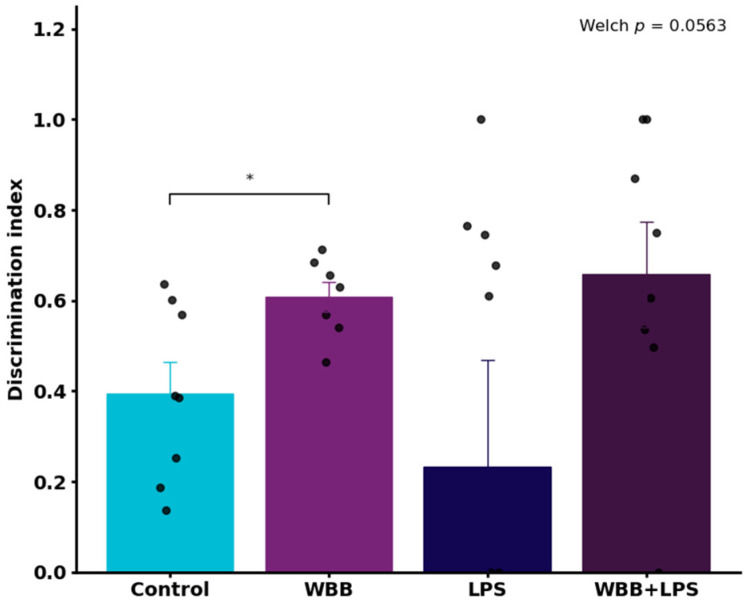
Discrimination index measured by the novel object recognition test. Data are presented as mean ± standard deviation. The asterisk denotes the pairwise difference between the Control and WBB groups identified by post hoc analysis (*p* < 0.05). Statistical analysis was performed using Welch’s ANOVA followed by Dunnett’s post hoc test. Experimental groups: Control (water), WBB (WBB-After formulation), LPS (water followed by lipopolysaccharide), and WBB+LPS (WBB-After formulation followed by LPS administration).

Behavioral performance was evaluated using the NOR test, which assesses short-term recognition memory (Figure 1). Welch’s ANOVA indicated a marginal overall effect among groups (*p* = 0.0563). Pairwise comparisons suggested a higher discrimination index in the WBB group compared with the Control group (*p* < 0.05).

### 3.5. Structural Integrity, Neuronal Damage, and Apoptosis in the Hippocampal CA1 Region

#### 3.5.1. Cytoarchitecture and Neuronal Density in the Hippocampal CA1 Region

**Figure 2:**
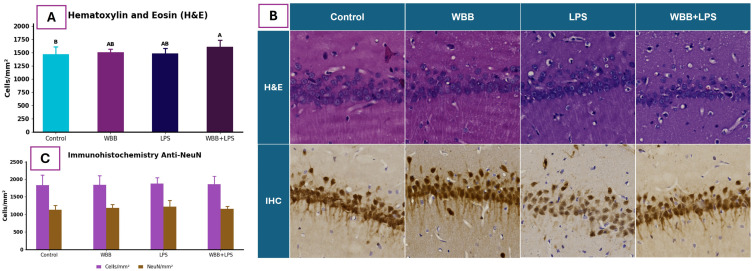
Histological and immunohistochemical analysis of the hippocampal CA1 region. (**A**) Quantification of total cell density (cells/mm^2^) in H&E-stained sections across experimental groups. Data are expressed as mean ± standard deviation. Different letters indicate statistically significant differences among groups (Tukey’s test, *p* < 0.05). (**B**) Representative micrographs of CA1 sections stained with H&E (upper row) and NeuN immunohistochemistry (IHC; lower row) for each group. Scale bar: 250 µm. (**C**) Quantification of total cells (cells/mm^2^) and NeuN-positive neurons (NeuN/mm^2^) in the CA1 region following immunohistochemical staining. Data are expressed as mean ± standard deviation. No significant differences were observed among groups (Tukey post hoc test, *p* > 0.05). Experimental groups: Control (water), WBB (WBB-After formulation), LPS (water followed by lipopolysaccharide), and WBB+LPS (WBB-After formulation followed by LPS administration). H&E: hematoxylin and eosin; IHC: immunohistochemistry; NeuN: neuronal nuclei; LPS: lipopolysaccharide.

Hematoxylin and eosin (H&E) staining revealed differences in total cell density (cells/mm^2^) within the CA1 region among experimental groups (Figure 2A). The WBB+LPS group exhibited a higher cell density compared to the Control group, whereas WBB and LPS showed intermediate values without significant differences relative to either group.

Representative micrographs (Figure 2B) showed a well-preserved cytoarchitecture of the CA1 pyramidal layer across all experimental groups. The pyramidal layer remained clearly defined, with no evident structural alterations among treatments.

Quantitative analysis of NeuN immunostaining indicated no significant differences in neuronal density (NeuN-positive cells/mm^2^) among groups (Figure 2C).

#### 3.5.2. Neuronal Apoptosis in the Hippocampal CA1 Region

**Figure 3:**
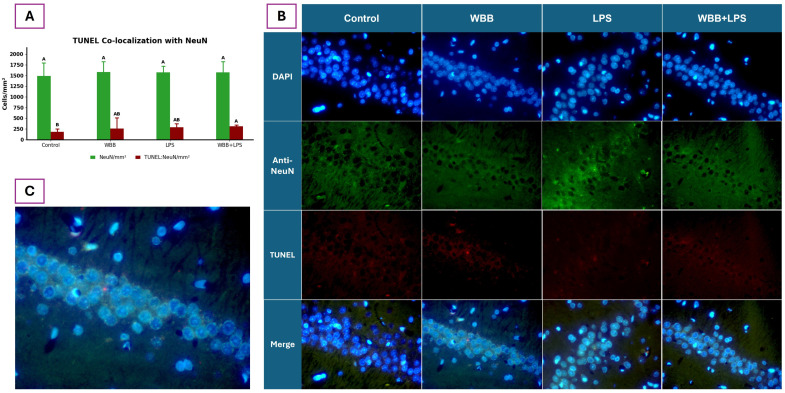
Neuronal apoptosis in the hippocampal CA1 region. (**A**) Quantification of NeuN-positive neurons (cells/mm^2^) and TUNEL-positive/NeuN-positive neurons (cells/mm^2^) across experimental groups. Data are expressed as mean ± standard deviation. Different letters indicate statistically significant differences among groups within each marker (Tukey’s test, *p* < 0.05). (**B**) Representative fluorescence micrographs of CA1 sections stained with DAPI (blue), NeuN (green), and TUNEL (red), along with merged images for each experimental group. (**C**) Higher-magnification merged image corresponding to the WBB group. Scale bar: 222 × 166.4 µm. Experimental groups: Control (water), WBB (WBB-After formulation), LPS (water followed by lipopolysaccharide), and WBB+LPS (WBB-After formulation followed by LPS administration). DAPI: 4′,6-diamidino-2-phenylindole; NeuN: neuronal nuclei; TUNEL: terminal deoxynucleotidyl transferase dUTP nick end labeling; LPS: lipopolysaccharide.

Apoptotic neurons in the hippocampal CA1 region were identified by TUNEL staining, co-localized with NeuN and quantified within defined ROIs (Figure 3A). NeuN-positive neuronal density remained stable across all experimental groups, with no significant differences detected (*p* > 0.05), indicating preserved neuronal presence at the analyzed time point. In contrast, the density of TUNEL-positive/NeuN-positive neurons showed a treatment-dependent pattern. The Control group exhibited significantly lower levels of apoptotic neurons compared to the WBB+LPS group, while the WBB and LPS groups showed intermediate values without significant differences relative to either group.

Representative fluorescence micrographs (Figure 3B) support these findings. DAPI nuclear staining, NeuN immunofluorescence, and TUNEL labeling confirmed the presence of apoptotic signals within NeuN-positive neurons. Increased TUNEL signal was observed in LPS-treated groups, particularly in the WBB+LPS condition, whereas Control sections showed minimal apoptotic labeling.

### 3.6. Hippocampal Cytokine Profile

**Figure 4:**
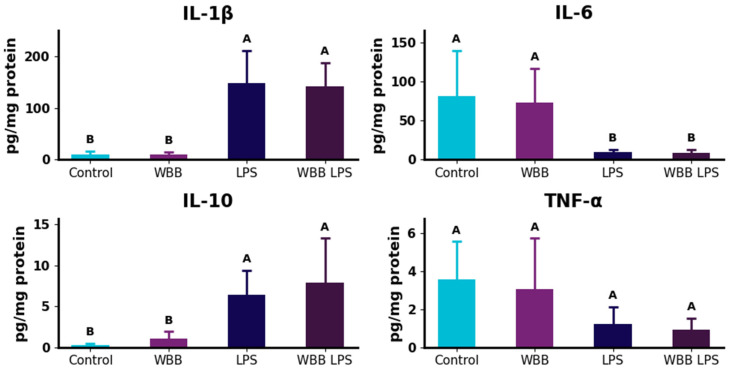
Cytokine levels in hippocampal tissue. Data are presented as mean ± standard deviation. Different letters indicate statistically significant differences among groups determined by Kruskal–Wallis analysis followed by Dunn’s multiple comparison test (*p* < 0.05). Experimental groups: Control (water), WBB (WBB-After formulation), LPS (water followed by lipopolysaccharide), and WBB+LPS (WBB-After formulation followed by LPS administration). IL: interleukin; TNF-α: tumor necrosis factor alpha; LPS: lipopolysaccharide.

Hippocampal cytokine levels showed distinct patterns depending on treatment (Figure 4). IL-1β concentrations were significantly increased in the LPS and WBB+LPS groups compared to Control and WBB, with no differences within either pair. IL-6 levels followed an opposite trend, with Control and WBB showing significantly higher values than LPS and WBB+LPS, and no differences within groups sharing the same statistical classification. TNF-α concentrations did not differ significantly among experimental groups. In contrast, IL-10 levels were significantly higher in the LPS and WBB+LPS groups compared to the Control and WBB, with no differences within either pair.

## 4. Discussion

### 4.1. Development and Baseline Characteristics of WBB Formulations

The development of food formulations with potential psychobiotic microorganisms requires matrices capable of supporting both microbial viability and metabolic activity [37]. In this context, whey and blueberry bagasse represent promising substrates from both functional and sustainability perspectives, enabling the valorization of agro-industrial by-products while serving as fermentation matrices [12,14]. In this study, a control formulation based on fermented whey alone and two additional formulations incorporating blueberry bagasse at different stages (before or after fermentation) were designed, allowing evaluation of how process timing influences fermentation performance and matrix properties. Under these conditions, whey—alone or supplemented with blueberry bagasse—effectively supported the growth of *L. plantarum* 299v and PS128, reaching cell densities above 8.6 log CFU/mL, within the range associated with potential health benefits [6].

All formulations exhibited a marked decrease in pH, reflecting active lactic acid fermentation. The higher titratable acidity observed in WBB-Before suggests that the presence of blueberry bagasse during fermentation may reflect increased microbial metabolic activity due to the availability of additional fermentable substrates provided by the bagasse during fermentation, as reported for fruit pomace fermentations [38]. This also contributed to lower soluble solids, indicating greater substrate utilization compared to WBB-After [39]. Acidification further supports microbiological stability by inhibiting spoilage and potentially pathogenic microorganisms [40]. Color differences among formulations were primarily driven by the incorporation of blueberry bagasse, with enriched samples showing reduced lightness and increased red tones. Variations in the b* coordinate, particularly the shift toward blue hues in WBB-Before, may be associated with anthocyanin transformations during fermentation [41], highlighting the impact of processing conditions on pigment stability.

From a compositional perspective, all formulations exhibited characteristics consistent with whey-based beverage-like systems, with high moisture and low fat and protein levels [42]. The incorporation of blueberry bagasse introduced dietary fiber, which was absent in the control formulation, confirming its role as a functional ingredient. Similar whey-based fermented systems enriched with berry by-products have been previously developed, supporting their potential as functional carriers in food applications [31].

### 4.2. Phenolic Composition of WBB Formulations and Its Functional Relevance

The incorporation of blueberry bagasse was the main determinant of the phenolic profile of WBB formulations, since no phenolic compounds were detected in WBB-Control. This confirms that the polyphenolic composition, and consequently a substantial part of the antioxidant and anti-inflammatory potential, was derived exclusively from the plant matrix. Consistent with the process variable introduced in Section 4.1, the stage of bagasse incorporation markedly influenced both total phenolic content and the distribution of individual compounds. Post-fermentation addition of blueberry bagasse favored phenolic preservation, whereas co-fermentation promoted transformation and altered extraction dynamics.

At the level of individual compounds, WBB-Before was enriched in flavan-3-ols and flavonols, likely due to fermentation-induced release of matrix-bound compounds [43,44,45]. In contrast, WBB-After showed higher levels of anthocyanins and hydroxycinnamic acids, including chlorogenic and syringic acids, suggesting greater preservation of these compounds when bagasse was added after [46,47]. The preservation of anthocyanins such as peonidin-3-glucoside and malvidin-3-glucoside is particularly relevant, as these compounds have been associated with antioxidant, anti-inflammatory, and potential neuroprotective effects in previous studies, including modulation of inflammatory signaling and protection against neuroinflammation [48,49,50]. Similarly, the higher retention of chlorogenic and syringic acids in WBB-After may contribute to antioxidant and anti-inflammatory responses, as suggested in previous studies [51,52]. Conversely, the presence of gallic acid and higher levels of proanthocyanidins in WBB-Before suggest active microbial transformation and enhanced release from the plant matrix during fermentation [53]. The detection of catechin and epicatechin only in WBB-After further supports their degradation or utilization during fermentation, consistent with the metabolic capacity of *L. plantarum* [54].

### 4.3. Functional Properties, GABA Production, and Selection of the Formulation for In Vivo Evaluation

The compositional differences between WBB formulations were directly reflected in their functional properties. As expected, WBB-Control showed minimal antioxidant activity, whereas both bagasse-containing formulations exhibited markedly higher ABTS and DPPH values, consistent with previous studies on blueberry by-products [14,27,55]. Although ABTS values were similar, WBB-After showed higher DPPH levels, suggesting that the preservation of specific phenolics, particularly anthocyanins and hydroxycinnamic acids, may be associated with a greater contribution to radical scavenging capacity. A similar trend was observed for anti-inflammatory capacity, where NO-RSA was not detected in WBB-Control and was highest in WBB-After. This response may reflect the combined effect of preserved blueberry polyphenols and bioactive peptides generated during whey fermentation, which have been associated with the modulation of inflammatory pathways and immune responses [18,56,57,58]. Nevertheless, these chemical assays primarily serve as screening tools and do not directly reflect biological effects in vivo.

In parallel, both bagasse-containing formulations exhibited enhanced GABA production compared to the control, reflecting the metabolic activity of the selected strains. Although WBB-Before showed slightly higher levels, WBB-After maintained concentrations comparable to those reported in other fermented matrices [31], within ranges associated with potential physiological effects [59,60,61]. In the present study, the estimated GABA intake corresponded to approximately 24.2 mg/100 mL for WBB-Before and 20.1 mg/100 mL for WBB-After, placing both formulations within the range proposed for functional foods.

GABA production is highly strain-dependent and influenced by substrate availability, fermentation conditions, and microbial interactions [62,63,64]. In this context, glutamate supplementation may have contributed to GABA synthesis, as its conversion via the glutamate decarboxylase pathway is a key metabolic route in LAB [62]. However, production in real food matrices is typically lower than in optimized media due to environmental and compositional constraints, highlighting the importance of matrix design [65]. Accordingly, variations in GABA levels among formulations may also be associated with matrix-related effects; however, as temporal dynamics were not evaluated, the underlying mechanism cannot be established from the present data. Residual glutamate was not quantified, which precludes estimation of conversion efficiency and limits interpretation of differences in GABA levels between formulations. From a functional perspective, GABA bioaccessibility may also be matrix-dependent, with values in whey-based LAB systems ranging from ~40–80% depending on the formulation [31,66], suggesting that the effective bioavailable fraction may differ from the total content measured.

The combined use of *L. plantarum* 299v and PS128 further supports the neurobiological potential of the formulation. Beyond GABA production, both strains have been associated with mechanisms related to the microbiota–gut–brain axis, including modulation of inflammation, neurotransmitter systems, and stress-related pathways [67,68,69,70,71], which may contribute to a broader functional spectrum than single-strain systems.

Taken together, these results indicate that the functional potential of the formulations may arise from the combined contribution of microbial activity, neuroactive metabolites, and plant-derived phenolics potentially interacting within the microbiota–gut–brain. Although WBB-Before showed favorable features, WBB-After provided the most balanced overall profile, combining higher phenolic content, a profile enriched in bioactive phenolics, enhanced antioxidant and anti-inflammatory activity, and GABA levels within a physiologically relevant range. This highlights the relevance of considering multi-component functional matrices, rather than single bioactive compounds, when designing food-based interventions targeting neurobiological processes. Future studies should compare formulations with distinct bioactive profiles to better elucidate their relative contributions.

### 4.4. Effects of WBB Administration on Recognition Memory in a Murine Model

The NOR task is widely used to assess recognition memory in rodents, based on their innate tendency to explore novel stimuli over familiar ones [72]. As it relies on spontaneous exploratory behavior rather than training, it is considered a robust method to detect subtle changes in cognitive performance [73]. Neurobiologically, NOR performance depends on the integrity of the perirhinal cortex and hippocampal circuits involved in recognition and declarative memory processing [74].

In this study, a murine model of systemic inflammation induced by LPS was used to evaluate the potential neurobiological effects of WBB. Animals exposed to LPS showed a tendency toward lower discrimination indices, suggesting a trend toward impaired recognition memory associated with inflammation, consistent with previous reports indicating that LPS can affect hippocampal function and cognitive performance [75]. However, no statistically significant differences were observed among LPS-treated groups under the conditions evaluated, indicating that no clear protective effect of WBB was detected under inflammatory challenge. In contrast, under basal conditions, animals receiving WBB showed higher discrimination indices compared with control animals, with a significant difference between these groups (*p* < 0.05), suggesting a possible effect on recognition memory. However, these findings should be interpreted with caution, as behavioral outcomes derived from a single test do not provide robust evidence of neurobiological effects, and further validation using complementary behavioral and molecular endpoints is required.

Behavioral outcomes in NOR tests are inherently variable and depend on multiple experimental factors, including strain selection, intervention duration, and model severity. In particular, the effects of microbial or fermentation-derived interventions on recognition memory are often modest and context-dependent [76]. However, some studies have reported improvements in NOR performance following probiotic or fermentation-based interventions, often associated with reduced oxidative stress, attenuation of neuroinflammation, and preservation of neuronal integrity [20,74]; such effects are not consistently observed across models and experimental conditions. These observations further highlight the importance of integrating behavioral outcomes with additional endpoints to better characterize the neurobiological potential of such formulations.

### 4.5. Hippocampal Structural Integrity and Neuronal Apoptosis

The hippocampus is a key brain region involved in learning and memory and is particularly sensitive to inflammatory and oxidative insults that can compromise neuronal integrity [77,78]. Accordingly, LPS-induced systemic inflammation is commonly used to model neuroinflammatory responses associated with cognitive dysfunction [75,79].

In the present study, hippocampal structural integrity in the CA1 region was evaluated using H&E staining and immunohistochemical approaches. No significant differences were observed in NeuN-positive neuronal density among groups (*p* > 0.05), indicating preserved neuronal presence under the experimental conditions. In contrast, neuronal apoptosis analysis showed increased TUNEL-positive/NeuN-positive cells in the WBB+LPS group compared with the Control (*p* < 0.05), while WBB and LPS groups exhibited intermediate values without significant differences. Importantly, no differences were observed between the WBB+LPS and LPS groups, suggesting that the increase in apoptotic labeling is primarily associated with the inflammatory challenge rather than WBB supplementation.

Although LPS has been widely reported to induce neuronal damage in the hippocampus [75,79], the lack of significant differences between the LPS-only and Control groups suggests that, under the conditions evaluated, LPS alone did not produce detectable neuronal apoptosis. This may be related to the timing between LPS administration and tissue collection, as the extent of neuronal damage is known to be time-dependent [75,79]. In parallel, H&E staining revealed increased cellularity in the WBB+LPS group compared to Control, although no differences were observed relative to the LPS group. As this method does not distinguish between neuronal and non-neuronal cells, the increase likely reflects infiltration or proliferation of glial or inflammatory cells rather than changes in neuronal density. The absence of differences between WBB+LPS and LPS groups further suggests that WBB supplementation did not further increase the inflammatory response under the conditions evaluated.

Microbial-derived interventions have been reported to modulate neuroimmune signaling and hippocampal structure. For example, probiotic administration has been associated with changes in hippocampal proteomic profiles and anti-inflammatory signaling [80], as well as improved neuronal organization and reduced degeneration [81,82]. Similarly, psychobiotic preparations from fermented foods have been reported to exert neuroprotective effects in previous studies [20]. However, no clear neuroprotective effects were observed under the conditions of the present study. It is also important to note that glial activation was not directly assessed; subtle changes in astrocyte or microglial activity may have occurred without detectable neuronal loss, potentially influencing the local inflammatory environment. Future studies incorporating specific glial markers are needed to clarify these mechanisms.

### 4.6. Hippocampal Cytokine Responses to LPS Challenge and WBB Supplementation

LPS administration is widely used to induce neuroinflammation through activation of innate immune signaling pathways, including Toll-like receptor signaling, downstream inflammatory responses, leading to the production of pro-inflammatory cytokines such as IL-1β in the hippocampus [83]. In addition, LPS has been reported to activate glial cells, further amplifying inflammatory signaling and inducing metabolic adaptations that support the energetic demands of neuroinflammation [83].

In the present study, LPS administration resulted in detectable alterations in hippocampal cytokine levels, confirming the induction of a neuroinflammatory response. Specifically, IL-1β and IL-10 were increased in LPS-treated groups, whereas IL-6 showed a decrease, and TNF-α remained unchanged. No significant differences were observed between LPS and WBB+LPS groups, indicating that WBB supplementation did not significantly modulate cytokine responses under the conditions evaluated. The differential behavior of cytokines may suggest a selective rather than uniform inflammatory response. The increase in IL-1β is consistent with canonical LPS-induced activation of inflammasome-related pathways [84], while the rise in IL-10 may indicate a compensatory anti-inflammatory response [85]. In contrast, changes in IL-6 and TNF-α likely reflect differences in regulatory pathways and temporal dynamics. Cytokine production is controlled by partially independent signaling cascades involving multiple signaling pathways, whose activation is time-dependent and can vary across conditions [86,87,88]. In addition, inflammatory responses may involve a transient activation followed by regulatory adjustments, leading to non-linear cytokine profiles [89].

Previous studies have reported anti-inflammatory effects of berry-derived bioactive compounds and fermented blueberry bagasse in cellular systems, including reductions in nitric oxide, reactive oxygen species, and cytokine production [18]. Blueberry anthocyanins have also been associated with modulation of oxidative stress and inflammatory signaling in neural tissues [50]. However, the absence of significant cytokine modulation in the present in vivo model suggests that these effects may not directly translate to complex physiological conditions.

Indeed, variability in cytokine responses across studies has been widely reported and may reflect differences in experimental design, including species, LPS dose, sampling time, and tissue analyzed. For example, stronger inflammatory responses have been reported in circulation than in brain tissue following LPS administration, with limited changes observed in hippocampal cytokines [90]. In addition, intervention-related factors such as duration and microbial dose may influence outcomes, as studies reporting psychobiotic effects typically employ longer treatment periods or higher microbial loads [68,69,71]. In the present study, animals were pre-treated with the formulation prior to LPS administration to assess its capacity to mitigate an acute neuroinflammatory challenge. Under this preventive framework, the absence of significant changes in cytokine levels suggests that no clear protective effect was detected under the conditions tested, which may also reflect the intensity of the LPS-induced response. It is important to note that the LPS dose used in this study (15 mg/kg) corresponds to endotoxic shock models (LD75) [32], which induce a robust acute inflammatory response. This may limit the detection of subtle modulatory effects, and results should be interpreted within this context.

More broadly, psychobiotic effects reported in the literature remain highly heterogeneous, with several studies describing modest or non-significant inflammatory changes depending on strain selection and experimental conditions [75]. Furthermore, the modulation of cytokines is often inconsistent across models and influenced by strain-specific responses [91,92]. In this context, the in vivo responses observed in the present study are aligned with the variability reported in the field.

Overall, these findings indicate that while LPS induced a neuroinflammatory response, WBB supplementation neither exacerbated nor attenuated cytokine expression in the hippocampus under the conditions tested. This outcome may reflect factors related to the experimental design, including intervention duration, dosage, and the specific endpoints evaluated, highlighting the complexity of translating in vitro functionality into in vivo outcomes, and that the mechanistic interpretations discussed are based on previously reported literature rather than direct assessment in the present study. In this context, it is important to consider that the in vivo evaluation was conducted using the selected formulation as an integrated matrix, and therefore, the observed effects reflect its overall composition rather than the contribution of individual components.

## 5. Conclusions

This study developed and characterized whey-based fermented formulations enriched with blueberry bagasse and evaluated their functional properties and explored their neurobiological potential through combined in vitro and in vivo approaches. The results demonstrated that the timing of bagasse incorporation significantly influenced the composition and functionality of the formulations, with post-fermentation addition favoring the preservation of bioactive phenolics and leading to enhanced antioxidant and anti-inflammatory properties. In addition, the formulations exhibited physiologically relevant GABA levels, supporting their potential as sustainable fermentation-based functional foods.

Under the experimental conditions evaluated, the selected formulation (WBB-After) showed a modest improvement in recognition performance under non-inflammatory conditions. In contrast, no measurable effects were observed under LPS-induced inflammation in behavioral, neuroinflammatory, or neuronal integrity markers. Importantly, these findings do not allow conclusions regarding the superiority of incorporation strategies at the neurobiological level, as only the WBB-After formulation was evaluated in vivo, and the observed neurobiological effects were limited and should be interpreted with caution. Future studies incorporating longer intervention periods, dose–response approaches, and expanded evaluation of microbiota–gut–brain axis interactions will be necessary to further elucidate its functional potential.

## Figures and Tables

**Table 1:** Microbial counts, physicochemical properties, and nutritional composition of whey biotic blend (WBB) formulations.

Parameters	WBB-Control	WBB-Before	WBB-After
Microbial counts (log CFU/mL)	8.67 ± 0.32 ^A^	9.02 ± 0.29 ^A^	8.66 ± 0.31 ^A^
pH	3.82 ± 0.04 ^A^	3.56 ± 0.01 ^B^	3.50 ± 0.05 ^B^
Titratable acidity (g lactic acid/100 g)	7.85 ± 0.21 ^B^	12.88 ± 0.26 ^A^	6.10 ± 0.23 ^C^
Soluble solids (°Brix)	6.90 ± 0.01 ^C^	7.40 ± 0.01 ^B^	7.90 ± 0.10 ^A^
Color parameters			
L*	90.19 ± 1.23 ^A^	33.01 ± 0.94 ^B^	31.26 ± 3.22 ^B^
a*	−2.83 ± 0.04 ^B^	19.55 ± 0.15 ^A^	18.97 ± 1.40 ^A^
b*	8.63 ± 0.11 ^A^	−1.70 ± 0.18 ^C^	5.17 ± 0.54 ^B^
Proximate composition (%)			
Moisture	93.09 ± 0.00 ^A^	91.76 ± 0.05 ^B^	91.70 ± 0.04 ^B^
Ash	0.49 ± 0.03 ^A^	0.32 ± 0.05 ^B^	0.28 ± 0.03 ^B^
Fat	0.37 ± 0.03 ^C^	0.66 ± 0.04 ^B^	1.13 ± 0.05 ^A^
Protein	0.89 ± 0.01 ^B^	1.01 ± 0.03 ^A^	1.00 ± 0.01 ^A^
Total carbohydrates	5.18 ± 0.01 ^C^	6.27 ± 0.01 ^A^	5.91 ± 0.03 ^B^
Dietary fiber	<LOD	1.15 ± 0.04 ^A^	1.18 ± 0.04 ^A^
Soluble dietary fiber	<LOD	0.13 ± 0.02 ^A^	0.12 ± 0.02 ^A^
Insoluble dietary fiber	<LOD	1.02 ± 0.08 ^A^	1.06 ± 0.09 ^A^

Values are expressed as mean ± standard deviation. Different uppercase letters within the same row indicate significant differences among formulations (Tukey’s test, *p* < 0.05). WBB-Control: fermented whey without blueberry bagasse; WBB-Before: whey with blueberry bagasse added before fermentation; WBB-After: whey with blueberry bagasse added after fermentation. Abbreviations: CFU: colony-forming units; LOD: limit of detection.

**Table 2:** Phenolic composition of WBB-Before and WBB-After formulations.

Compound (µg/g d.w.) ^1^	WBB-Before	WBB-After
Hydroxybenzoic acids		
Gallic acid	115.97 ± 6.38	ND
Syringic acid	78.98 ± 9.67 ^B^	190.24 ± 27.90 ^A^
Hydroxycinnamic acids		
Chlorogenic acid	35.12 ± 0.29 ^B^	427.60 ± 2.75 ^A^
Caffeic acid	ND	24.73 ± 0.30
Caffeic acid derivative	195.65 ± 11.38 ^A^	28.99 ± 0.16 ^B^
Caffeoylquinic acid derivative	376.05 ± 5.43 ^A^	38.07 ± 1.14 ^B^
*p*-Coumaric acid	ND	27.53 ± 0.27
Flavan-3-ols		
Proanthocyanidins ^2^	343.72 ± 20.8 ^A^	29.26 ± 0.10 ^B^
Catechin	ND	49.28 ± 0.19
Epicatechin	ND	31.83 ± 0.08
Flavonols		
Quercetin-3-glucoside	43.06 ± 0.41 ^A^	28.89 ± 0.30 ^A^
Quercetin-3-galactoside	74.47 ± 45.66 ^A^	19.06 ± 0.35 ^B^
Quercetin-3-arabinoside	122.53 ± 4.78 ^A^	28.71 ± 3.75 ^B^
Quercetin (aglycone)	16.49 ± 4.38 ^A^	1.08 ± 0.02 ^B^
Isorhamnetin	22.79 ± 0.41 ^A^	30.83 ± 1.98 ^A^
Kaempferol	63.50 ± 4.68 ^A^	62.88 ± 6.90 ^A^
Myricetin glycoside	65.57 ± 4.70 ^A^	2.36 ± 0.10 ^B^
Anthocyanins		
Cyanidin-3-glucoside	ND	290.02 ± 1.54
Delphinidin-3-arabinoside	233.66 ± 0.07 ^A^	218.29 ± 0.64 ^A^
Petunidin-3-galactoside	ND	163.00 ± 0.20
Petunidin-3-arabinoside	ND	128.30 ± 0.56
Peonidin-3-glucoside	25.15 ± 4.44 ^B^	490.96 ± 3.05 ^A^
Malvidin-3-galactoside	27.54 ± 0.26 ^B^	71.47 ± 0.21 ^A^
Malvidin-3-glucoside	96.54 ± 4.02 ^B^	293.84 ± 1.76 ^A^
Malvidin-3-arabinoside	36.94 ± 0.62 ^B^	61.00 ± 1.69 ^A^
Acylated anthocyanin	29.35 ± 0.47 ^B^	92.64 ± 0.23 ^A^
Spectrophotometric determination		
TPC (mg GAE/g d.w.)	3.85 ± 0.33 ^B^	8.17 ± 0.82 ^A^

^1^ Individual phenolic compounds are expressed as µg/g dry weight (d.w.), whereas total phenolic content (TPC) is expressed as mg gallic acid equivalents (GAE)/g d.w. ^2^ Proanthocyanidins were quantified as grouped compounds, including dimeric and oligomeric forms. Values are presented as mean ± standard deviation. Different uppercase letters within the same row indicate significant differences between formulations (Student’s *t*-test, *p* < 0.05). WBB-Before: whey with blueberry bagasse added before fermentation; WBB-After: whey with blueberry bagasse added after fermentation. ND: not detected. Compound identification and retention times are provided in Table S1. Representative chromatograms are shown in Figures S1 and S2.

**Table 3:** Antioxidant and anti-inflammatory capacities, and GABA content of WBB formulations.

Parameter ^1^	WBB-Control	WBB-Before	WBB-After
ABTS (mg TE/g)	0.36 ± 0.06 ^B^	2.44 ± 0.02 ^A^	2.46 ± 0.01 ^A^
DPPH (mg TE/g)	0.12 ± 0.04 ^C^	3.03 ± 0.12 ^B^	3.76 ± 0.04 ^A^
NO-RSA (mg TE/g)	ND	14.46 ± 1.27 ^B^	17.22 ± 0.81 ^A^
GABA (mM)	0.41 ± 0.03 ^C^	2.35 ± 0.05 ^A^	1.95 ± 0.19 ^B^

^1^ Antioxidant and anti-inflammatory capacities are expressed on a dry weight basis, whereas GABA concentrations were determined in fresh samples. Results are expressed as mean ± standard deviation. Different uppercase letters within the same row indicate significant differences among formulations (Tukey’s test, *p* < 0.05). WBB-Control: fermented whey without blueberry bagasse; WBB-Before: whey with blueberry bagasse added before fermentation; WBB-After: whey with blueberry bagasse added after fermentation. ABTS: 2,2′-azinobis(3-ethylbenzothiazoline-6-sulfonic acid) radical; DPPH: 2,2-diphenyl-1-picrylhydrazyl radical; NO-RSA: nitric oxide radical scavenging activity; TE: Trolox equivalents; GABA: γ-aminobutyric acid; ND: not detected.

## Data Availability

Data are contained within the article.

## Supplementary Materials

The following supporting information can be downloaded at: https://www.mdpi.com/article/10.3390/nu18101558/s1, Table S1: Ultra-high-performance liquid chromatography retention times and UV-Vis spectral characteristics of phenolic compounds identified in whey biotic blend formulations enriched with blueberry bagasse; Figure S1: Representative ultra-high-performance liquid chromatography chromatograms of whey biotic blend formulation with blueberry bagasse added before fermentation, recorded at 280, 360, and 520 nm; Figure S2: Representative ultra-high-performance liquid chromatography chromatograms of whey biotic blend formulation with blueberry bagasse added after fermentation, recorded at 280, 360, and 520 nm.

## Abbreviations

The following abbreviations are used in this manuscript:

ABTS	2,2′-azinobis(3-ethylbenzothiazoline-6-sulfonic acid)
ANOVA	Analysis of variance
AOAC	Association of Official Analytical Chemists
CFU	Colony-forming units
CIBO	Centro de Investigación Biomédica de Occidente
CIE	Commission Internationale de l’Éclairage
DAPI	4′,6-diamidino-2-phenylindole
DPPH	2,2-diphenyl-1-picrylhydrazyl
d.w.	Dry weight
GABA	γ-Aminobutyric acid
GAE	Gallic acid equivalents
H&E	Hematoxylin and eosin
HIER	Heat-induced epitope retrieval
IHC	Immunohistochemistry
IL	Interleukin
IL-1β	interleukin-1β
IL-6	interleukin-6
IL-10	interleukin-10
LAB	Lactic acid bacteria
LPS	Lipopolysaccharide
MRS	de Man, Rogosa and Sharpe
NADPH	Nicotinamide adenine dinucleotide phosphate, reduced form
NeuN	Neuronal nuclei
NO-RSA	Nitric oxide radical scavenging activity
PBS	Phosphate-buffered saline
PS128	*Lactiplantibacillus plantarum* PS128
ROI	Region of interest
TdT	Terminal deoxynucleotidyl transferase
TE	Trolox equivalents
TNF-α	Tumor necrosis factor alpha
TPC	Total phenolic content
TUNEL	Terminal deoxynucleotidyl transferase dUTP nick end labeling
UHPLC	Ultra-high-performance liquid chromatography
UV-Vis	Ultraviolet visible
WBB	Whey Biotic Blend

## References

[1] World Health Organization Mental Disorders. https://www.who.int/news-room/fact-sheets/detail/mental-disorders.

[2] Moitra M., Santomauro D., Collins P.Y., Vos T., Whiteford H., Saxena S., Ferrari A.J. (2022). The global gap in treatment coverage for major depressive disorder in 84 countries from 2000–2019: A systematic review and Bayesian meta-regression analysis. PLoS Med..

[3] Carding S., Verbeke K., Vipond D.T., Corfe B.M., Owen L.J. (2015). Dysbiosis of the gut microbiota in disease. Microb. Ecol. Health Dis..

[4] Yoo J.Y., Groer M., Dutra S.V.O., Sarkar A., McSkimming D.I. (2020). Gut microbiota and immune system interactions. Microorganisms.

[5] Balasubramanian R., Schneider E., Gunnigle E., Cotter P.D., Cryan J.F. (2024). Fermented foods: Harnessing their potential to modulate the microbiota-gut-brain axis for mental health. Neurosci. Biobehav. Rev..

[6] Del Toro-Barbosa M., Hurtado-Romero A., Garcia-Amezquita L.E., García-Cayuela T. (2020). Psychobiotics: Mechanisms of action, evaluation methods and effectiveness in applications with food products. Nutrients.

[7] Liu Y.-W., Liu W.-H., Wu C.-C., Juan Y.-C., Wu Y.-C., Tsai H.-P., Wang S., Tsai Y.-C. (2016). Psychotropic effects of *Lactobacillus plantarum* PS128 in early life-stressed and naïve adult mice. Brain Res..

[8] Rudzki L., Ostrowska L., Pawlak D., Małus A., Pawlak K., Waszkiewicz N., Szulc A. (2019). Probiotic *Lactobacillus plantarum* 299v decreases kynurenine concentration and improves cognitive functions in patients with major depression: A double-blind, randomized, placebo-controlled study. Psychoneuroendocrinology.

[9] Ochoa-de la Paz L.D., Gulias-Cañizo R., D’Abril Ruíz-Leyja E., Sánchez-Castillo H., Parodí J. (2021). The role of GABA neurotransmitter in the human central nervous system, physiology, and pathophysiology. Rev. Mex. Neurocienc..

[10] Fashogbon R.O., Samson O.J., Awotundun T.A., Olanbiwoninu A.A., Adebayo-Tayo B.C. (2024). Microbial gamma-aminobutyric acid synthesis: A promising approach for functional food and pharmaceutical applications. Lett. Appl. Microbiol..

[11] Rocha-Mendoza D., Kosmerl E., Krentz A., Zhang L., Badiger S., Miyagusuku-Cruzado G., Mayta-Apaza A., Giusti M., Jiménez-Flores R., García-Cano I. (2021). Invited review: Acid whey trends and health benefits. J. Dairy Sci..

[12] Uribe-Velázquez T., Díaz-Vázquez D., Barajas-Álvarez P., González-López M.E., Gradilla-Hernández M.S., Garcia-Amezquita L.E., Carrillo-Nieves D., García-Cayuela T. (2025). From waste to value: Mitigating the environmental impact of whey in Jalisco, Mexico. J. Clean. Prod..

[13] Klavins L., Kviesis J., Nakurte I., Klavins M. (2018). Berry press residues as a valuable source of polyphenolics: Extraction optimisation and analysis. LWT.

[14] Hurtado-Romero A., Garcia-Amezquita L.E., Carrillo-Nieves D., Montilla A., Villamiel M., Requena T., García-Cayuela T. (2024). Characterization of berry by-products as fermentable substrates: Proximate and phenolic composition, antimicrobial activity, and probiotic growth dynamics. LWT.

[15] Kumar A., Saranyadevi S., Thirumalaisamy S.K., Dapana Durage T.T., Jaiswal S.G., Kavitake D., Wei S. (2025). Phenolic Acids in Fermented Foods: Microbial Biotransformation, Antioxidant Mechanisms, and Functional Health Implications. Front. Mol. Biosci..

[16] de Wolf R.X.M., Hider R.N., Breitmeyer J., Serventi L. (2025). Effect of Lactic Fermentation and Matrix on Phenolic Content, Bioaccessibility, and Scavenging Activity of Beetroot Beverages. Eur. Food Res. Technol..

[17] Gao B., Wang J., Wang Y., Xu Z., Li B., Meng X., Sun X., Zhu J. (2022). Influence of Fermentation by Lactic Acid Bacteria and In Vitro Digestion on the Biotransformations of Blueberry Juice Phenolics. Food Control.

[18] Hurtado-Romero A., Luzardo-Ocampo I., Antunes-Ricardo M., López-Pacheco F., Garcia-Amezquita L.E., Carrillo-Nieves D., García-Cayuela T. (2026). Fungal solid-state and submerged fermentation of blueberry bagasse: Extraction strategies, phenolic profiling, and cellular immunomodulation. Fermentation.

[19] Sivapragasam N., Neelakandan N., Rupasinghe H.P.V. (2023). Potential Health Benefits of Fermented Blueberry: A Review of Current Scientific Evidence. Trends Food Sci. Technol..

[20] Luang-In V., Katisart T., Konsue A., Nudmamud-Thanoi S., Narbad A., Saengha W., Wangkahart E., Pumriw S., Samappito W., Ma N.L. (2020). Psychobiotic effects of multi-strain probiotics originated from Thai fermented foods in a rat model. Food Sci. Anim. Resour..

[21] (2012). Leche—Denominaciones, Especificaciones Fisicoquímicas, Información Comercial y Métodos de Prueba.

[22] (1994). Determinación de Humedad en Alimentos por Tratamiento Térmico. Método por Arena o Gasa.

[23] (2020). Alimentos—Determinación de Cenizas en Alimentos—Método de Prueba.

[24] (1994). Alimentos y Bebidas no Alcohólicas Con modificaciones en su Composición. Especificaciones Nutrimentales.

[25] (2011). Alimentos—Determinación de Proteínas en Alimentos—Método de Ensayo (Prueba).

[26] Garcia-Amezquita L.E., Tejada-Ortigoza V., Serna-Saldivar S.O., Welti-Chanes J. (2018). Dietary Fiber Concentrates from Fruit and Vegetable By-Products: Processing, Modification, and Application as Functional Ingredients. Food Bioprocess Technol..

[27] Hurtado-Romero A., Zepeda-Hernández A., Cárdenas-Rangel J., Aguilar-Márquez R., Garcia-Amezquita L.E., Carrillo-Nieves D., García-Cayuela T. (2025). Frozen fermented dairy snacks with probiotics and blueberry bagasse: Stability, bioactivity, and digestive viability. Microorganisms.

[28] Sanchez-Rangel J.C., Benavides J., Heredia J.B., Cisneros-Zevallos L., Jacobo-Velazquez D.A. (2013). The Folin–Ciocalteu assay revisited: Improvement of its specificity for total phenolic content determination. Anal. Methods.

[29] Méndez-Galarraga M.P., Hurtado-Romero A., Pirovani M.E., Vinderola G., Van De Velde F., García-Cayuela T. (2023). Exploring autochthonous strains with probiotic potential: A comprehensive characterization of functional properties and their application in fermented blueberry-watermelon smoothies. Food Biosci..

[30] Patel A., Patel A., Patel A., Patel N.M. (2010). Determination of polyphenols and free radical scavenging activity of *Tephrosia purpurea* Linn leaves (Leguminosae). Pharmacogn. Res..

[31] Del Toro-Barbosa M., Uribe-Velázquez T., Hurtado-Romero A., Rosales-De la Cruz M.F., Carrillo-Nieves D., Garcia-Amezquita L.E., García-Cayuela T. (2025). Evaluation of GABA-producing fermented whey formulations: From strain selection to raspberry-enriched beverages with psychobiotic potential. Foods.

[32] Ramírez-Jirano L.J., Zenteno-Savín T., Gaxiola-Robles R., Ramos-González E.J., Torres-Mendoza B.M., Bitzer-Quintero O.K. (2016). The Neuroprotective Effect of Erythropoietin in Rat Hippocampus in an Endotoxic Shock Model. Rev. Investig. Clin..

[33] Martínez-Torres N.I., Cárdenas-Bedoya J., Torres-Mendoza B.M. (2024). Acute combined cerebrolysin and nicotinamide administration promote cognitive recovery through neuronal changes in the hippocampus of rats with permanent middle cerebral artery occlusion. Neuroscience.

[34] Bankhead P., Loughrey M.B., Fernández J.A., Dombrowski Y., McArt D.G., Dunne P.D., McQuaid S., Gray R.T., Murray L.J., Coleman H.G. (2017). QuPath: Open source software for digital pathology image analysis. Sci. Rep..

[35] Paxinos G., Watson C. (2007). The Rat Brain in Stereotaxic Coordinates.

[36] Gaidica M. Rat Brain Atlas (Interactive Atlas). https://labs.gaidi.ca/rat-brain-atlas/.

[37] Cichońska P., Kowalska E., Ziarno M. (2023). The survival of psychobiotics in fermented food and the gastrointestinal tract: A review. Microorganisms.

[38] Kalinowska M., Gołębiewska E., Zawadzka M., Choińska R., Koronkiewicz K., Piasecka-Jóźwiak K., Bujak M. (2023). Sustainable extraction of bioactive compounds from apple pomace through lactic acid bacteria (LAB) fermentation. Sci. Rep..

[39] Thuy C.X., Pham V.T., Nguyen T.T.N.H., Nguyen T.T.N., Ton N.T.A., Tuu T.T., Vu N.D. (2024). Effect of fermentation conditions (dilution ratio, medium pH, total soluble solids, and *Saccharomyces cerevisiae* yeast ratio) on the ability to ferment cider from tamarillo (*Solanum betaceum*) fruit. J. Food Process. Preserv..

[40] Zapaśnik A., Sokołowska B., Bryła M. (2022). Role of lactic acid bacteria in food preservation and safety. Foods.

[41] Yuan K., Wu G., Li X., Zeng Y., Wen X., Liu R., Jiang X., Tian L., Sun J., Bai W. (2024). Anthocyanins degradation mediated by β-glycosidase contributes to the color loss during alcoholic fermentation in a structure-dependent manner. Food Res. Int..

[42] Chuck-Hernandez C., García-Cayuela T., Méndez-Merino E. (2022). Dairy-Based Snacks. Snack Foods.

[43] Le Bourvellec C., Bagano Vilas Boas P., Lepercq P., Comtet-Marre S., Auffret P., Ruiz P., Bott R., Schols H.A., Renard C.M.G.C., Mosoni P. (2019). Procyanidin–cell wall interactions within apple matrices decrease the metabolization of procyanidins by the human gut microbiota and the anti-inflammatory effect of the resulting microbial metabolome in vitro. Nutrients.

[44] Zhong Y., Zhang J., Zhao Q., Manickam S., Wu Y., Han Y., Shao S., Tao Y. (2026). Fermentation of blueberry slurry by lactobacilli strains: Investigation on soluble and insoluble fractions. Food Chem..

[45] Cheng S., Zheng Y., Li H., Jiang Y., Zhang Y. (2025). Metabolic engineering of blueberry juice by *Lactiplantibacillus plantarum* JM065: Fermentation optimization. Food Chem..

[46] Fritsch C., Jänsch A., Ehrmann M.A., Toelstede S., Vogel R.F. (2017). Characterization of cinnamoyl esterases from different *Lactobacilli* and *Bifidobacteria*. Curr. Microbiol..

[47] Yang M., Zhang M., Wang B., Zhang Z., Zhuang Y., Liu J., Zhang Q., Fei P. (2025). Mechanism-driven stabilization of anthocyanins: Comparative copigmentation and encapsulation for food applications. Food Chem..

[48] Carey A.N., Fisher D.R., Rimando A.M., Gomes S.M., Bielinski D.F., Shukitt-Hale B. (2013). Stilbenes and anthocyanins reduce stress signaling in BV-2 mouse microglia. J. Agric. Food Chem..

[49] Huang W.Y., Fu L., Li C.Y., Xu L.P., Zhang L.X., Zhang W.M. (2017). Quercetin, hyperin, and chlorogenic acid improve endothelial function by antioxidant, anti-inflammatory, and ACE inhibitory effects. J. Food Sci..

[50] Wang L., Lan W., Chen D. (2024). Blueberry (*Vaccinium* spp.) anthocyanins and their functions, stability, bioavailability, and applications. Foods.

[51] Liu H., Meng H., Du M., Lv H., Wang Y., Zhang K. (2024). Chlorogenic acid ameliorates intestinal inflammation by inhibiting NF-κB and endoplasmic reticulum stress in lipopolysaccharide-challenged broilers. Poult. Sci..

[52] Olszowy-Tomczyk M. (2024). Factors influencing the antioxidant properties of binary mixtures of quercetin and chlorogenic acid as well as quercetin and kaempferol. Chem. Pap..

[53] Dhiman S., Mukherjee G. (2022). Biovalorization of fruit waste to gallic acid synthesis through the application of *Bacillus haynesii* SSRY4 MN031245 tannase. Mater. Today Proc..

[54] Pulido-Mateos E.C., Lessard-Lord J., Desjardins Y., Roy D. (2024). *Lactiplantibacillus plantarum* interstrain variability in the production of bioactive phenolic metabolites from flavan-3-ols. J. Agric. Food Chem..

[55] Rojas-Ocampo E., Torrejón-Valqui L., Muñóz-Astecker L.D., Medina-Mendoza M., Mori-Mestanza D., Castro-Alayo E.M. (2021). Antioxidant capacity, total phenolic content and phenolic compounds of pulp and bagasse of four Peruvian berries. Heliyon.

[56] Zeng X., Wang Y., Yang S., Liu Y., Li X., Liu D. (2023). The functionalities and applications of whey/whey protein in fermented foods: A review. Food Sci. Biotechnol..

[57] Méndez-Galarraga M.P., Hurtado-Romero A., Antunes-Ricardo M., Garcia-Amezquita L.E., Pirovani M.E., Vinderola G., Van De Velde F., García-Cayuela T. (2025). Enhancing safety and bioactivity of blueberry-watermelon smoothies through combined ultrasound and lactic acid fermentation with potential probiotics. Food Biosci..

[58] Sar T., Bogovic Matijasic B., Danilovic B., Gamero A., Gandía M., Krausova G., Martínez-Villaluenga C., Peñas E., Bagherzadehsurbagh E., Cemali Ö. (2025). A systematic review of health promoting effects of consumption of whey-based fermented products on adults. Front. Nutr..

[59] Dutta S.D., Patel D.K., Ganguly K., Lim K.-T. (2021). Effects of GABA/β-glucan supplements on melatonin and serotonin content extracted from natural resources. PLoS ONE.

[60] Nakamura H., Takishima T., Kometani T., Yokogoshi H. (2009). Psychological stress-reducing effect of chocolate enriched with γ-aminobutyric acid (GABA) in humans: Assessment of stress using heart rate variability and salivary chromogranin A. Int. J. Food Sci. Nutr..

[61] Okada T., Sugishita T., Murakami T., Murai H., Saikusa T., Horino T., Onoda A., Kajimoto O., Takahashi R., Takahashi T. (2000). Effect of the defatted rice germ enriched with GABA for sleeplessness, depression, and autonomic disorder by oral administration. Nippon Shokuhin Kagaku Kogaku Kaishi.

[62] Montagano F., Prete R., Fanti F., Dell’Orco F., Oliva E., Compagnone D., Corsetti A. (2025). Enrichment of γ-aminobutyric acid (GABA) in a legume-based beverage through the fermentation by *Lactiplantibacillus plantarum*. Curr. Res. Food Sci..

[63] Wonglapsuwan M., Ninrat T., Chaichana N., Dechathai T., Suwannasin S., Singkhamanan K., Pomwised R., Surachat K. (2025). Global genomic landscapes of *Lactiplantibacillus plantarum*: Universal GABA biosynthetic capacity with strain-level functional diversity. Life.

[64] Xu R., Mayer M.J., Philo M., Gall G.L., Mulaw G., Ponsero A., Narbad A. (2026). Combining *Lactiplantibacillus plantarum* and *Bifidobacterium adolescentis* can improve GABA production in faecal fermentations. J. Appl. Microbiol..

[65] Hou D., Tang J., Feng Q., Niu Z., Shen Q., Wang L., Zhou S. (2024). Gamma-aminobutyric acid (GABA): A comprehensive review of dietary sources, enrichment technologies, processing effects, health benefits, and its applications. Crit. Rev. Food Sci. Nutr..

[66] Uribe-Velázquez T., Zepeda-Hernández A., Luzardo-Ocampo I., Rosales-de la Cruz M.F., Garcia-Amezquita L.E., Carrillo-Nieves D., García-Cayuela T. (2026). Bioactive Properties and GABA Production of Whey-, Rice-, and Oat-Based Fermented Formulations under Simulated Gastrointestinal Digestion. J. Agric. Food Res..

[67] Kaźmierczak-Siedlecka K., Folwarski M., Skonieczna-Żydecka K., Ruszkowski J., Makarewicz W. (2020). The use of *Lactobacillus plantarum* 299v (DSM 9843) in cancer patients receiving home enteral nutrition—Study protocol for a randomized, double-blind, and placebo-controlled trial. Nutr. J..

[68] Raff H., Hainsworth K.R., Woyach V.L., Weihrauch D., Wang X., Dean C. (2024). Probiotic and high-fat diet: Effects on pain assessment, body composition, and cytokines in male and female adolescent and adult rats. Am. J. Physiol. Regul. Integr. Comp. Physiol..

[69] Ma Y.-F., Lin Y.-A., Huang C.-L., Hsu C.-C., Wang S., Yeh S.-R., Tsai Y.-C. (2023). *Lactiplantibacillus plantarum* PS128 alleviates exaggerated cortical beta oscillations and motor deficits in the 6-hydroxydopamine rat model of Parkinson’s disease. Probiotics Antimicrob. Proteins.

[70] Lee M.-C., Lin T.-A., Huang C.-C. (2025). Dual-strain psychobiotics combining live *Lactiplantibacillus plantarum* PS128 and heat-treated *Lacticaseibacillus paracasei* PS23 improve psychological and neuroendocrine outcomes in stressed adults: A randomized, placebo-controlled trial. Foods.

[71] Lee Y.Z., Cheng S.-H., Chang M.-Y., Lin Y.-F., Wu C.-C., Tsai Y.-C. (2023). Neuroprotective effects of *Lactobacillus plantarum* PS128 in a mouse model of Parkinson’s disease: The role of gut microbiota and microRNAs. Int. J. Mol. Sci..

[72] Mathiasen J.R., DiCamillo A. (2010). Novel object recognition in the rat: A facile assay for cognitive function. Curr. Protoc. Pharmacol..

[73] Wooden J.I., Spinetta M.J., Nguyen T., O’Leary C.I., Leasure J.L. (2021). A sensitive homecage-based novel object recognition task for rodents. Front. Behav. Neurosci..

[74] Grayson B., Leger M., Piercy C., Adamson L., Harte M., Neill J.C. (2015). Assessment of disease-related cognitive impairments using the novel object recognition (NOR) task in rodents. Behav. Brain Res..

[75] Zhao J., Bi W., Xiao S., Lan X., Cheng X., Zhang J., Lu D., Wei W., Wang Y., Li H. (2019). Neuroinflammation induced by lipopolysaccharide causes cognitive impairment in mice. Sci. Rep..

[76] Sarkar A., Lehto S.M., Harty S., Dinan T.G., Cryan J.F., Burnet P.W.J. (2016). Psychobiotics and the manipulation of bacteria–gut–brain signals. Trends Neurosci..

[77] Perosa V., Zanon Zotin M.C., Schoemaker D., Sveikata L., Etherton M.R., Charidimou A., Greenberg S.M., Viswanathan A. (2024). Association between hippocampal volumes and cognition in cerebral amyloid angiopathy. Neurology.

[78] Jung H., Lee D., You H., Lee M., Kim H., Cheong E., Um J.W. (2023). LPS induces microglial activation and GABAergic synaptic deficits in the hippocampus accompanied by prolonged cognitive impairment. Sci. Rep..

[79] Batey L., Baumberger B., Khoshbouei H., Hashemi P. (2024). Lipopolysaccharide effects on neurotransmission: Understanding implications for depression. ACS Chem. Neurosci..

[80] Loupy K.M., Dawud L.M., Zambrano C.A., Lee T., Heinze J.D., Elsayed A.I., Hassell J.E., D’Angelo H.M., Frank M.G., Maier S.F. (2025). Effects of oral administration of the probiotic *Lactobacillus rhamnosus* GG on the proteomic profiles of cerebrospinal fluid and immunoregulatory signaling in the hippocampus of adult male rats. Neuroimmunomodulation.

[81] Lin J.-Y., Tsai B.C.-K., Kao H.-C., Chiang C.-Y., Chen Y.-A., Chen W.S.-T., Ho T.-J., Yao C.-H., Kuo W.-W., Huang C.-Y. (2023). Neuroprotective effects of probiotic *Lactobacillus reuteri* GMNL-263 in the hippocampus of streptozotocin-induced diabetic rats. Probiotics Antimicrob. Proteins.

[82] Yu X., Yu X., Yang Y., Cheng W., Shi M., Chen L., Zhang X., Xu Y. (2025). Probiotic Bifico ameliorates depression- and anxiety-like behaviors induced by estrogen deficiency via NLRP3 inflammasome inhibition. J. Inflamm. Res..

[83] Vizuete A.F.K., Fróes F., Seady M., Zanotto C., Bobermin L.D., Roginski A.C., Wajner M., Quincozes-Santos A., Gonçalves C.A. (2022). Early effects of LPS-induced neuroinflammation on the rat hippocampal glycolytic pathway. J. Neuroinflamm..

[84] Garlanda C., Di Ceglie I., Jaillon S. (2025). IL-1 family cytokines in inflammation and immunity. Cell. Mol. Immunol..

[85] Branchett W.J., Saraiva M., O’Garra A. (2024). Regulation of inflammation by interleukin-10 in the intestinal and respiratory mucosa. Curr. Opin. Immunol..

[86] Luecke S., Guo X., Sheu K.M., Singh A., Lowe S.C., Han M., Díaz J., Lopes F., Wollman R., Hoffmann A. (2024). Dynamical and combinatorial coding by MAPK p38 and NF-κB in the inflammatory response of macrophages. Mol. Syst. Biol..

[87] Daniels M.A., Teixeiro E. (2025). The NF-κB signaling network in the life of T cells. Front. Immunol..

[88] Park B.S., Lee J.O. (2013). Recognition of lipopolysaccharide pattern by TLR4 complexes. Exp. Mol. Med..

[89] Medzhitov R. (2008). Origin and physiological roles of inflammation. Nature.

[90] Carregosa D., Loncarevic-Vasiljkovic N., Feliciano R., Moura-Louro D., Mendes C.S., dos Santos C.N. (2024). Locomotor and gait changes in the LPS model of neuroinflammation are correlated with inflammatory cytokines in blood and brain. J. Inflamm..

[91] Cryan J.F., O’Riordan K.J., Cowan C.S.M., Sandhu K.V., Bastiaanssen T.F.S., Boehme M., Codagnone M.G., Cussotto S., Fulling C., Golubeva A.V. (2019). The microbiota–gut–brain axis. Physiol. Rev..

[92] Dantzer R., O’Connor J.C., Freund G.G., Johnson R.W., Kelley K.W. (2008). From inflammation to sickness and depression: When the immune system subjugates the brain. Nat. Rev. Neurosci..

