# Supplementary material for: Blueberry Bagasse-Enriched Whey Fermented Formulations: Effect of Incorporation Timing on Functional Properties and Neurobiological Evaluation in a Murine Model Using a Selected Formulation
Source: Nutrients. 2026 May 14;18(10):1558. doi: 10.3390/nu18101558 (PMC13209441; doi:10.3390/nu18101558)
Supplement: Supplementary file 1 [file nutrients-18-01558-s001.zip › nutrients-4278018-supplementary.pdf]

nutrients

MDPI

Supplementary Materials

Blueberry Bagasse-Enriched Whey Fermented Formulations: Effect of Incorporation Timing on Functional Properties and Neurobiological Evaluation in a Murine Model Using a Selected Formulation

Tlalli Uribe-Velázquez [1], Alejandra Hurtado-Romero [1], Juliana Marisol Godínez-Rubí [2], Oscar Kurt Bitzer-Quintero [3], Javier Ramírez-Jirano [3], Félix Tadeo Ortiz-Sánchez [3], Jhonathan Cárdenas-Bedoya [3,4], Pablo Quintero-Gutiérrez [1], Iván Luzardo-Ocampo [1,5], Angélica Lizeth Sánchez-López [1], Luis Eduardo García-Amezquita [6], Danay Carrillo-Nieves [1], and Tomás García-Cayuela [1,*]

1 Tecnologico de Monterrey, School of Engineering and Sciences, Ave. General Ramón Corona 2514, 45138 Zapopan, Jal., Mexico; a01633168@tec.mx (T.U-V.); ale.hr@tec.mx (A.H.-R.); A01644533@tec.mx (P.Q.-G.); ivanluzardo@tec.mx (I.L.-O.); alsl@tec.mx (A.L.S.-L.); danay.carrillo@tec.mx (D.C-N.)

2 Laboratorio de Patología Diagnóstica e Inmunohistoquímica, Centro de Investigación y Diagnóstico de Patología, Departamento de Microbiología y Patología, Centro Universitario de Ciencias de la Salud, Universidad de Guadalajara, Guadalajara 44340, Jal., Mexico; juliana.godinez@academicos.udg.mx (J.M.G.-L)

3 Instituto Mexicano del Seguro Social, Centro de Investigación Biomédica de Occidente, División de Neurociencias, Guadalajara 44340, Jal., Mexico; neuronim26@yahoo.com (O.K.B.-Q.); ramirez_jirano@hotmail.com (J.R.-J.); felixtadeo2010@gmail.com (F.T.O.-S.); jhonathan.cbedoya@academicos.udg.mx (J.C.-B.)

4 Departamento de Disciplinas Filósofico, Metodológicas e Instrumentales, Centro Universitario de Ciencias de la Salud, Universidad de Guadalajara, Guadalajara 44340, Jal., Mexico.

5 Tecnologico de Monterrey, Institute for Obesity Research, Ave. Eugenio Garza Sada 2501 Sur, 64849 Monterrey, N.L., Mexico.

6 Tecnologico de Monterrey, School of Engineering and Sciences, Ave. Eugenio Garza Sada 2501 Sur, 64849 Monterrey, N.L., Mexico; garcia-amezquita@tec.mx (L.E.G.-A.).

* Correspondence: tomasgc@tec.mx (T.G.-C.)

Materials:

Table S1. Ultra-high-performance liquid chromatography retention times and UV-Vis spectral characteristics of phenolic compounds identified in whey biotic blend formulations enriched with blueberry bagasse.

Figure S1. Representative ultra-high-performance liquid chromatography chromatograms of whey biotic blend formulation with blueberry bagasse added before fermentation, recorded at 280, 360, and 520 nm.

Figure S2. Representative ultra-high-performance liquid chromatography chromatograms of whey biotic blend formulation with blueberry bagasse added after fermentation, recorded at 280, 360, and 520 nm.

Table S1. Ultra-high-performance liquid chromatography retention times and UV-Vis spectral characteristics of phenolic compounds identified in whey biotic blend formulations enriched with blueberry bagasse.

**Peak No.***	**Retention time (min)**	**λ_{max} (nm)**	**Compound**
1	3.085	271	Gallic acid [a]
2	3.762	284	Procyanidin dimer [b]
3	3.933	327	Caffeic acid [b]
4	5.970	288	Procyanidin oligomer [b]
5	6.406	274	Procyanidin dimer [b]
6	6.921	341	Caffeic acid derivative [b]
7	7.380	278	Procyanidin dimer [b]
8	8.770	284	Catechin [a]
9	9.542	325	Chlorogenic acid [a]
10	10.537	521	Cyanidin-3-glucoside [a]
11	12.557	280	Epicatechin [a]
12	13.259	326	Caffeoylquinic acid derivative [b]
13	14.634	521	Delphinidin-3-arabinoside [a]
14	16.578	345	p-Coumaric acid [a]
15	17.325	523.8	Petunidin-3-galactoside [a]
16	20.070	523.8	Petunidin-3-arabinoside [b]
17	21.099	527.5	Peonidin-3-glucoside [b]
18	22.067	526.3	Malvidin-3-galactoside [b]
19	22.831	527.5	Malvidin-3-glucoside [a]
20	23.028	528.7	Malvidin-3-arabinoside [b]
21	24.215	325, 532	Acylated anthocyanin [b]
22	25.308	273	Syringic acid [b]
23	25.908	354.6	Quercetin-3-glucoside [b]
24	26.460	354.6	Quercetin-3-galactoside [b]
25	27.293	354.6	Quercetin-3-arabinoside [b]
26	27.878	352.2	Quercetin (aglycone) [a]
27	28.330	349.8	Isorhamnetin [b]
28	30.248	352	Kaempferol [a]
29	30.439	367.5	Myricetin glycoside [b]

* Peak numbers are according to Figs. S1 and S2.

[a] Compounds quantified using commercial standards.

[b] Compounds quantified using structurally related commercial standards.

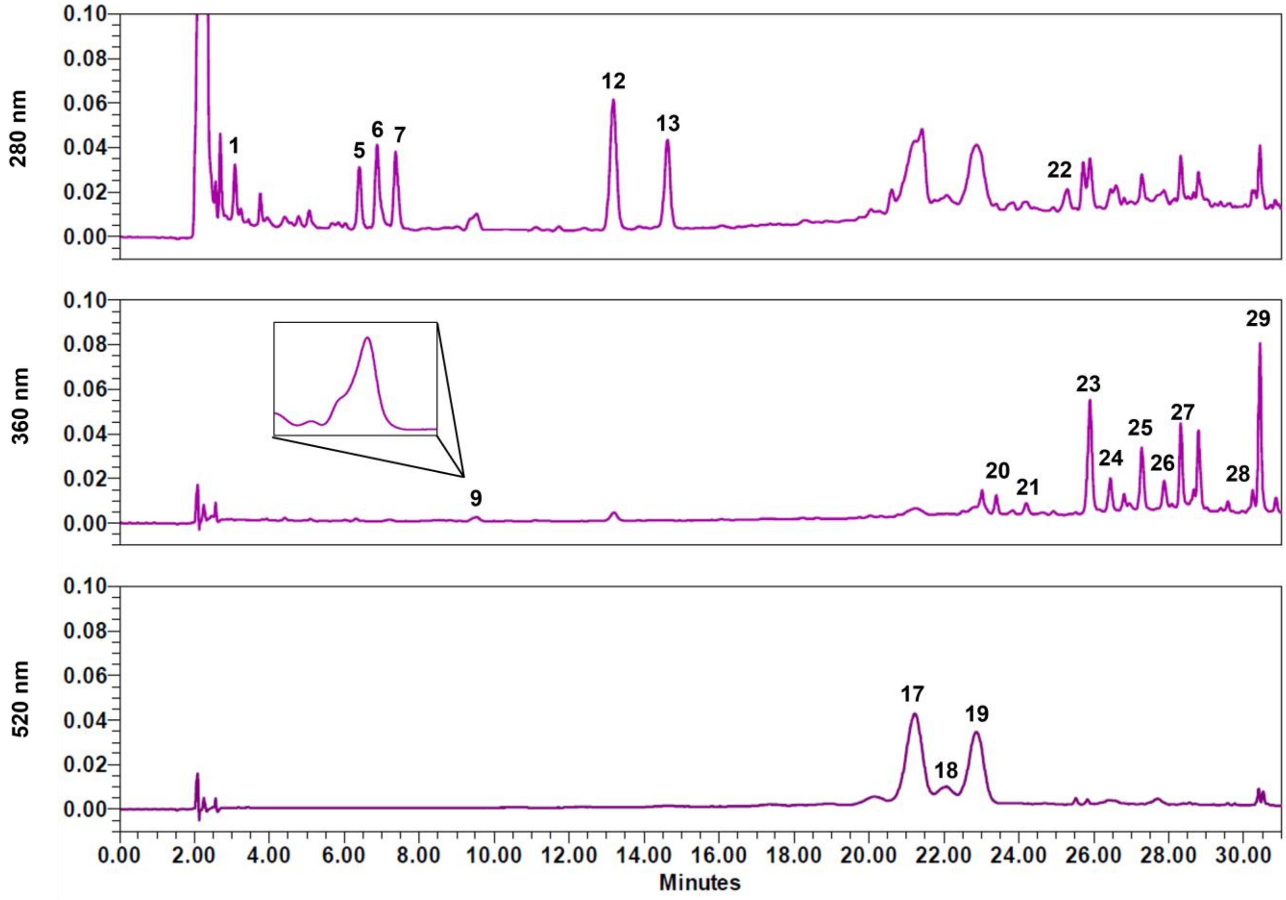

Figure S1. Representative ultra-high-performance liquid chromatography chromatograms of whey biotic blend formulation with blueberry bagasse added before fermentation, recorded at 280, 360, and 520 nm. Peak identities are provided in Table S1.

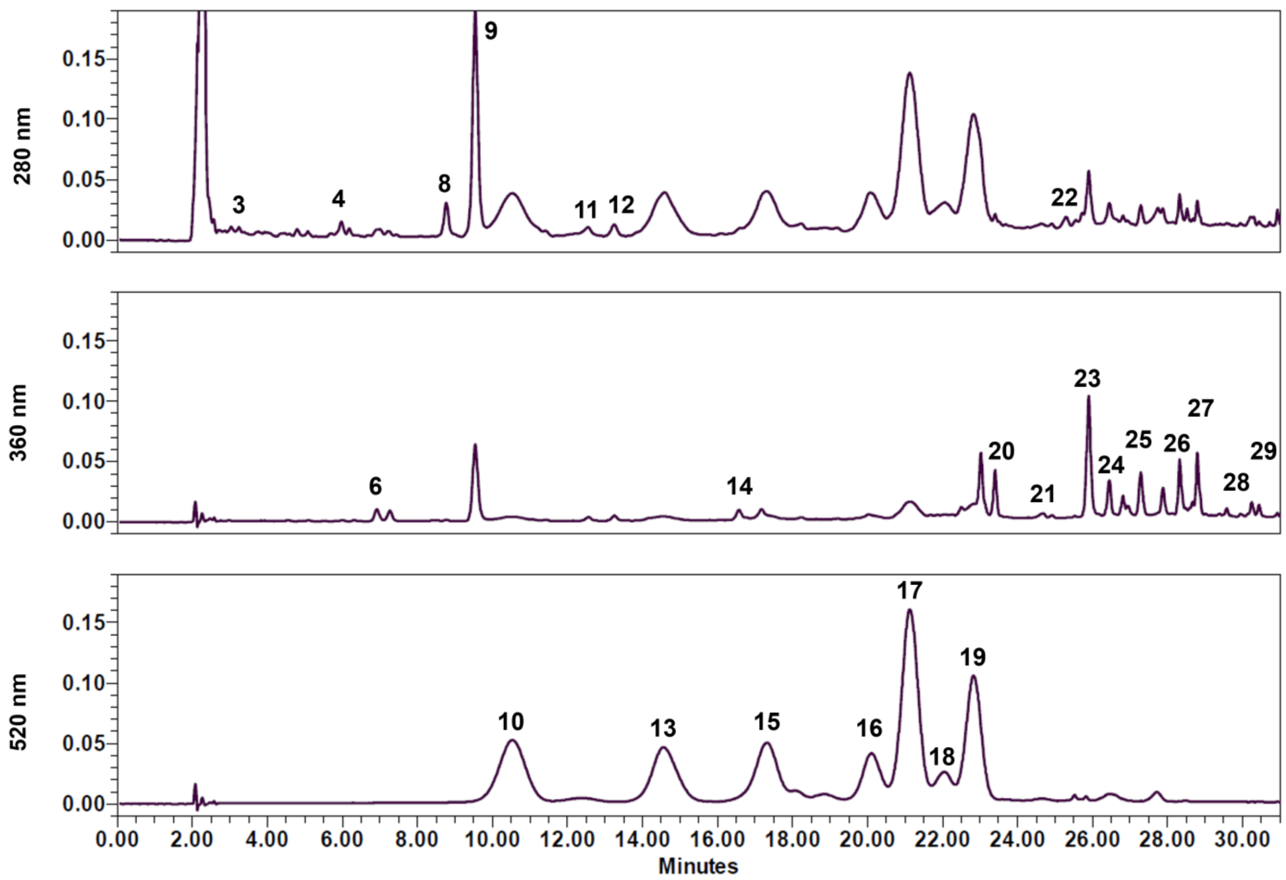

Figure S2. Representative ultra-high-performance liquid chromatography chromatograms of whey biotic blend formulation with blueberry bagasse added after fermentation, recorded at 280, 360, and 520 nm. Peak identities are provided in Table S1.
